# Do Glioblastoma Cancer Cells Need Vitamins? The Role of Vitamin B_1_

**DOI:** 10.3390/cancers18213423

**Published:** 2026-10-22

**Authors:** Jan Korbecki, Mateusz Bosiacki, Patrycja Kupnicka, Marta Tkacz, Maciej Tarnowski, Patrycja Kłos, Magdalena Szenejko, Magdalena Gąssowska-Dobrowolska, Krzysztof Safranow, Agnieszka Kolasa

**Affiliations:** 1 Institute of Health Sciences, Collegium Medicum, University of Zielona Gora, Zyty 28 St., 65-417 Zielona Gora, Poland; 2 Department of Biochemistry and Medical Chemistry, Pomeranian Medical University in Szczecin, Al. Powstańców Wlkp. 72, 70-111 Szczecin, Poland; mateusz.bosiacki@pum.edu.pl (M.B.); patrycja.kupnicka@pum.edu.pl (P.K.); patrycja.klos@pum.edu.pl (P.K.); krzysztof.safranow@pum.edu.pl (K.S.); 3 Department of Physiology in Health Sciences, Faculty of Health Sciences, Pomeranian Medical University in Szczecin, Zolnierska 48, 70-210 Szczecin, Poland; marta.tkacz@pum.edu.pl (M.T.);; 4 Institute of Marine and Environmental Sciences, University of Szczecin, Mickiewicza 16 St., 70-383 Szczecin, Poland; magdalena.szenejko@usz.edu.pl; 5 The Centre for Molecular Biology and Biotechnology, University of Szczecin, Wąska 13 St., 71-415 Szczecin, Poland; 6 Department of Cellular Signalling, Mossakowski Medical Research Institute, Polish Academy of Sciences, Pawińskiego 5, 02-106 Warsaw, Poland; 7 Department of Histology and Embryology, Faculty of Medicine, Pomeranian Medical University in Szczecin, Al. Powstańców Wielkopolskich 72, 70-111 Szczecin, Poland; agnieszka.kolasa@pum.edu.pl

**Keywords:** glioblastoma, thiamine, vitamin B_1_, oxythiamine, amprolium, temozolomide, brain tumor, thiamin pyrophosphokinase 1, thiamine antimetabolite

## Abstract

**Background/Objectives**: In this study, we investigated whether targeting thiamine (vitamin B_1_) metabolism could be useful in the treatment of glioblastoma. **Methods**: The study was conducted using LN-229 and T98G glioblastoma cell lines, as well as glioblastoma tissue samples obtained from up to 30 patients. The effects of thiamine and the thiamine antimetabolites oxythiamine and amprolium on cell viability were assessed using the MTT assay. The effects of hypoxia and temozolomide (TMZ), as well as gene expression in tissue samples, were analyzed using qRT-PCR. The expression of *SLC19A2*, *SLC19A3*, *TPK1*, *SLC25A19*, *SLC35F3*, and *SLC44A4* was evaluated. **Results**: Higher thiamine concentrations increased the MTT metabolic activity of glioblastoma cells. Oxythiamine and amprolium reduced MTT metabolic activity, although their inhibitory effects reached a plateau and neither compound enhanced the effect of TMZ. Chronic hypoxia reduces thiamine metabolism. The expression of proteins involved in thiamine metabolism is lower in glioblastoma tissue than in peritumoral tissue. **Conclusions**: Overall, targeting thiamine metabolism does not appear to improve the efficacy of TMZ-based glioblastoma treatment. However, the observed viability-promoting effect of higher thiamine concentrations indicates that excessive thiamine intake or supplementation in patients with glioblastoma should be approached with caution and requires further clinical investigation.

## Simple Summary

Glioblastoma is a brain tumor characterized by a poor prognosis. For this reason, glioblastoma is being intensively studied. Glioblastoma cells require vitamins. Therefore, this study aims to investigate the role of vitamin B_1_ (thiamine) in this type of cancer. Thiamine increased the MTT metabolic activity of glioblastoma cells. Thiamine antimetabolites (oxythiamine and amprolium) reduce glioblastoma cells’ MTT metabolic activity. Studies on glioblastoma cells have shown that hypoxia reduces vitamin B_1_ metabolism. Thiamine antimetabolites may have therapeutic potential against glioblastoma.

## 1. Introduction

Thiamine, also known as vitamin B_1_, is a water-soluble vitamin. The recommended dietary allowance (RDA) for thiamine is 1.2 mg/day for men and 1.1 mg/day for women [1]. The main dietary sources of thiamine include cereal products [2], legumes [3], and meat, particularly pork [4]. Thiamine deficiency remains a major problem in populations in developing countries [5]. By contrast, in developed countries, it is reported in only a few percent of the population [6,7]. It is particularly prevalent among individuals with alcohol use disorder [8], patients with diabetes [9], and people with eating disorders such as anorexia nervosa [10].

Once absorbed from the diet, thiamine enters the bloodstream and is subsequently taken up by cells mainly via the transporters solute carrier family 19 member 2 (SLC19A2)/THTR1 [11] and solute carrier family 19 member 3 (SLC19A3)/THTR2 [12]. SLC19A3 exhibits more favorable transport kinetics than SLC19A2 [13]. Other, less well-characterized thiamine transporters include solute carrier family 22 member 1 (SLC22A1)/OCT1, solute carrier family 22 member 2 (SLC22A2)/OCT2 [14], and solute carrier family 35 member 3 (SLC35F3) [15]. Solute carrier family 44 member 4 (SLC44A4) does not transport thiamine itself but instead transports thiamine pyrophosphate (TPP) and acetylcholine [16]. In the cytoplasm, thiamine is phosphorylated to TPP by thiamin pyrophosphokinase 1 (TPK1) [17]. TPP can subsequently be transported into the mitochondria by solute carrier family 25 member 19 (SLC25A19) [18]. TPP is the predominant intracellular form of thiamine, whereas unphosphorylated thiamine is the main form found in blood plasma [19].

TPP serves as a coenzyme for several enzymes. Thiamine deficiency or therapeutic interference with thiamine metabolism reduces the activity of TPP-dependent enzymes, including:

- Enzymes with transketolase activity, including transketolase (TKT) [20], transketolase-like protein 1 (TKTL1), and transketolase-like protein 2 (TKTL2) [21]. These enzymes function in the non-oxidative phase of the pentose phosphate pathway, in which glycolytic intermediates are converted into ribose 5-phosphate required for DNA and RNA synthesis [22];
- The pyruvate dehydrogenase complex (PDC) [23], which catalyzes the conversion of glycolysis-derived pyruvate into acetyl-CoA, which subsequently enters the tricarboxylic acid (TCA) cycle;
- 2-oxoglutarate dehydrogenase (OGDH) [24], an enzyme of the TCA cycle;
- Branched-chain α-keto acid dehydrogenase (BCKDH) [25], which participates in the catabolism of the branched-chain amino acids leucine, isoleucine, and valine;
- Dehydrogenase E1 and transketolase domain-containing protein 1 (DHTKD1) [26], which is involved in the catabolism of lysine, hydroxylysine, and tryptophan;
- 2-hydroxyacyl-CoA lyase 1 (HACL1) [27], which is responsible for the peroxisomal α-oxidation of phytanic acid.

Thiamine metabolism is closely associated with cancer, as cancer cells require vitamins, including thiamine, to maintain their growth and metabolic activity. Accordingly, dietary thiamine restriction has been shown to inhibit tumor growth in in vivo models of breast cancer [28,29]. Moreover, physiological blood thiamine concentrations appear to be below those that maximize tumor growth. In an in vivo model of Ehrlich ascites tumor, thiamine supplementation promoted tumor growth, with the strongest effect observed at a dose 25 times higher than the RDA [30]. At a dose 125 times higher than the RDA, tumor growth returned to the level observed in the absence of supplementation. At still higher, millimolar concentrations, thiamine inhibits the growth of cancer cells [30,31,32]. This effect has been associated with decreased pyruvate dehydrogenase kinase (PDK) activity and the consequent activation of PDC in cancer cells. However, the cytotoxic effects of such high thiamine concentrations may also explain these findings [33].

The relationship between thiamine availability and tumor growth may be explained by the metabolic functions of this vitamin. Thiamine is required for the activity of TKT [20], TKTL1, and TKTL2 [21]. These enzymes participate in the non-oxidative phase of the pentose phosphate pathway and contribute to the production of ribose 5-phosphate, a precursor required for DNA and RNA synthesis [22]. Thiamine is also essential for the activity of OGDH, an enzyme of the TCA cycle [24]. Through oxidative TCA-cycle metabolism, cancer cells can convert glutamine-derived 2-oxoglutarate into downstream metabolites, including oxaloacetate [34] and isocitrate [35]. Oxaloacetate can subsequently be converted into aspartate, which is used in the de novo synthesis of purines and pyrimidines. In some types of cancer, however, 2-oxoglutarate undergoes reductive carboxylation to form isocitrate and citrate, bypassing OGDH [35]. Citrate can then provide acetyl-CoA for fatty acid synthesis.

Glioblastoma is associated with an extremely poor prognosis. The current median survival of patients with glioblastoma is estimated to be approximately one year [36,37,38]. Consequently, the molecular and metabolic mechanisms underlying glioblastoma are being intensively investigated to identify new therapeutic targets and improve existing treatments. One potentially relevant aspect of glioblastoma biology is vitamin B_1_ metabolism, which may represent a therapeutic target. Therefore, the aim of the present study was to investigate the significance of thiamine in glioblastoma. The specific research questions were as follows:

- Is the viability of glioblastoma cells dependent on thiamine availability?
- Does therapeutic targeting of vitamin B_1_ metabolism affect the viability of glioblastoma cells?
- Is the response of glioblastoma cells to anticancer treatment associated with vitamin B_1_ metabolism?
- Is vitamin B_1_ metabolism in glioblastoma cells altered by the tumor microenvironment or anticancer treatment?

## 2. Materials and Methods

### 2.1. Cell Culture and Treatment

All in vitro experiments were performed using LN-229 (CRL-2611; American Type Culture Collection [ATCC], Manassas, VA, USA) and T98G (CRL-1690; ATCC) cells, obtained from ATCC through its distributor, LGC Standards (Warsaw, Poland). The cells were cultured in 25 cm^2^ cell culture flasks (707001; NEST, Wuxi, Jiangsu, China) and maintained in a humidified incubator at 37 °C with 5% CO_2_. Immediately after purchase, the cells were cultured in the media recommended by the manufacturer. LN-229 cells were cultured in Dulbecco′s modified eagle′s medium (DMEM) containing 4.5 g/L glucose and L-glutamine (11965-092; Gibco, Waltham, MA, USA), supplemented with penicillin at a final concentration of 100 U/mL, streptomycin at a final concentration of 100 µg/mL (15140-122; Gibco), and 10% heat-inactivated fetal bovine serum (FBS) (A52569-01; Gibco). Immediately after purchase, T98G cells were cultured in minimum essential medium (MEM) containing 1.0 g/L glucose (15-010-CV; Corning, Glendale, AZ, USA), supplemented with GlutaMAX (35050-061; Gibco) as a source of L-glutamine, non-essential amino acid solution (M7145-100ML; Merck, Darmstadt, Germany), penicillin and streptomycin (15140-122; Gibco), and 10% heat-inactivated FBS (A52569-01; Gibco). The cells were passaged twice weekly. They were first washed twice with Dulbecco’s phosphate-buffered saline (DPBS) without calcium or magnesium ions (14190-144; Gibco) and then incubated with trypsin–ethylenedinitrilotetraacetic acid (EDTA) solution (T4174; Merck) for 30–40 s. Cells were cultured up to passage 6 and then cryopreserved using Synth-a-Freeze™ Cryopreservation Medium (A1254201; Gibco) for subsequent experiments. Cells at passage 10 or later were used for experiments.

DMEM contains 11.87 µM thiamine, corresponding to 4.0 mg/L [39], whereas MEM contains 2.97 µM thiamine, corresponding to 1.0 mg/L [40]. By comparison, the plasma thiamine concentration is approximately 10 nM, corresponding to approximately 2.65 µg/L [19]. In cerebrospinal fluid (CSF), thiamine occurs predominantly as unphosphorylated thiamine and thiamine monophosphate, at concentrations of approximately 20 nM and 30 nM, respectively [19]. Thus, the culture media recommended by the manufacturers contain approximately 300-fold more thiamine in the case of MEM and approximately 1200-fold more thiamine in the case of DMEM than is present in plasma. These media were therefore considered unsuitable for investigating the role of vitamin B_1_ metabolism in cancer. For this reason, Medium 199, which contains only 29.67 nM thiamine [41], was used in the experiments. This medium is commonly used for in vitro studies of vitamin B_1_ metabolism [42,43]. Because cells accumulate thiamine, they were adapted to the experimental conditions by culturing them for one week, corresponding to two passages, in Medium 199 containing 1.0 g/L glucose and L-glutamine (CC121-0500; Simply biologics, Taoyuan, Taiwan), antibiotics (15140-122; Gibco), and 10% heat-inactivated FBS (A52569-01; Gibco). The cells were then seeded for the individual experiments.

A single bottle of FBS was used throughout all experiments to minimize differences in nutrient concentrations between serum batches. Based on published data, the thiamine concentration in FBS was estimated to be 122 nM [44], whereas Medium 199 contains 29.67 nM thiamine [41]. Thus, Medium 199 supplemented with 10% FBS, as used in the experiments, contained an estimated thiamine concentration of 38.9 nM. During the experimental period, Medium 199 supplemented with FBS did not significantly inhibit cell growth. At the end of the experiments, control cells cultured in this medium covered almost the entire bottom surface of the wells of the 96-well plates.

### 2.2. Effects of Thiamine and Its Antimetabolites on Glioblastoma Cell Viability

To investigate the effect of thiamine on glioblastoma cell viability, the cells were seeded in 96-well cell culture plates (701001; NEST) at a density of 700 cells/well in 100 µL of medium. Only cell suspensions with viability exceeding 95% were used in the experiments. Cell viability was assessed using trypan blue solution (T8154; Merck), and cell viability and cell numbers were determined using a TC20 automated cell counter (Bio-Rad, Hercules, CA, USA). After 6 h, a stock solution of thiamine hydrochloride, CAS No. 67-03-8 (T1270; Merck), was prepared in Medium 199 supplemented with antibiotics and 10% FBS. Thiamine was dissolved in the medium at a concentration of 8 mM and incubated at 37 °C for 10 min with mixing. The resulting solution was passed through a syringe filter with a pore size of 0.22 µm (99722; TPP, Trasadingen, Switzerland), diluted to the required concentration, and added to the cells in a volume of 100 µL. Freshly prepared thiamine solutions were used in all experiments. The final volume of medium in each well was 200 µL, and the final concentration of added thiamine ranged from 50 nM to 4 mM. In each independent experiment, every concentration was tested in triplicate on each plate, whereas the control condition was tested in 15 wells. After three days of incubation, the thiamine-containing medium was replaced. After a further three days, the experiment was terminated, and a 3-(4,5-dimethylthiazol-2-yl)-2,5-diphenyltetrazolium bromide (MTT) assay was performed. Five independent experiments were conducted for each cell line. Results were expressed relative to control cells, to which no additional thiamine had been added.

The cytotoxic effects of the vitamin B_1_ antimetabolites oxythiamine chloride hydrochloride, CAS No. 614-05-1 (O4000; Merck), and amprolium hydrochloride, CAS No. 137-88-2 (31592-250MG; Merck), were also investigated.

The anticancer properties of oxythiamine have been well characterized in numerous studies [43,45,46,47]. It is the most extensively studied vitamin B_1_ antimetabolite as a potential anticancer agent and was therefore selected for our experiments. Oxythiamine inhibits transketolase [30]. At high concentrations exceeding 100 μM, it inhibits thiamine uptake [48]. At a concentration of 4 mM, it also inhibits TPK1 activity [45].

Amprolium is used to treat parasitic infections in animals [49]. At a concentration of 10 μM, it inhibits cellular thiamine uptake [48]. At a much higher concentration of 180 μM, amprolium also inhibits TPK1 activity, thereby inhibiting thiamine metabolism [45]. It was selected as the second compound for our experiments because its mechanism of action differs from that of oxythiamine.

These compounds were selected because they are among the most extensively studied vitamin B_1_ antimetabolites in cancer research [43,45,46,47]. LN-229 and T98G cells were seeded in 96-well cell culture plates (701001; NEST) at a density of 2000 cells/well in 200 µL of Medium 199 supplemented with antibiotics and 10% FBS. After 48 h of incubation, solutions of the antimetabolites were prepared. Oxythiamine chloride hydrochloride and amprolium hydrochloride were dissolved in Medium 199 supplemented with 10% FBS at a concentration of 10 mM. After incubation at 37 °C for 10 min, the solutions were passed through a syringe filter with a pore size of 0.22 µm (99722; TPP). The medium was then removed from the cells and replaced with medium containing the respective antimetabolite. Freshly prepared antimetabolite solutions were used in all experiments. Oxythiamine concentrations ranging from 1 µM to 1 mM and amprolium concentrations ranging from 2 µM to 1 mM were tested. Each concentration was tested in duplicate on each plate. After 72 h of incubation, an MTT assay was performed. At least five independent experiments were conducted for each cell line. The results were expressed relative to untreated control cells cultured in the experimental medium.

### 2.3. Effects of Thiamine and Its Antimetabolites on the Anticancer Activity of Temozolomide

Temozolomide (TMZ) is the drug most commonly used to treat glioblastoma [50,51,52]. It was therefore selected for the present experiments. This part of the study was designed to determine whether thiamine and its antimetabolites affected the anticancer activity of TMZ. The effect of TMZ alone on the viability of LN-229 and T98G glioblastoma cells was examined first. A 50 mM TMZ stock solution was prepared by dissolving TMZ, CAS No. 85622-93-1 (T2577; Merck), in dimethyl sulfoxide (DMSO) (D2438; Merck) at a concentration of 9.70 mg/mL. LN-229 and T98G cells were seeded in 96-well cell culture plates (701001; NEST) at a density of 2000 cells/well in 200 µL of Medium 199 supplemented with antibiotics and 10% FBS. After 48 h of incubation, the medium was removed and replaced with medium containing TMZ at concentrations ranging from 10 to 500 µM. DMSO controls corresponding to the DMSO concentrations present in the 200 and 500 µM TMZ treatments were also included. The effects of TMZ on cell viability were expressed relative to the corresponding DMSO control. At lower TMZ concentrations, for which the DMSO concentration was negligible, results were expressed relative to cells cultured in experimental medium alone. Each TMZ concentration was tested in duplicate on each plate. After 72 h of incubation, an MTT assay was performed. At least five independent experiments were conducted for each cell line.

The effects of thiamine on the anticancer activity of TMZ were then investigated. Cells were seeded in 96-well cell culture plates (701001; NEST) at a density of 700 cells/well in 100 µL of Medium 199 supplemented with antibiotics and 10% FBS. After 6 h of incubation, 100 µL of a thiamine solution prepared in the experimental medium was added. The total volume of medium in each well was therefore 200 µL. Based on the results of the experiments examining the effect of thiamine on glioblastoma cell MTT metabolic activity, a final thiamine concentration of 2 µM was selected. After 48 h of incubation, the medium was removed and replaced with medium containing thiamine at a final concentration of 2 µM, TMZ at a final concentration of either 200 or 500 µM, or the respective combinations of these compounds. A DMSO control was also included. After 72 h of incubation, an MTT assay was performed. Each treatment condition was tested in four replicate wells. At least five independent experiments were conducted for each cell line.

The potential interaction between the anticancer effects of the vitamin B_1_ antimetabolites and TMZ was also investigated. Cells were seeded in 96-well cell culture plates (701001; NEST) at a density of 2000 cells/well in 100 µL of Medium 199 supplemented with antibiotics and 10% FBS. After 24 h of incubation, 100 µL of an oxythiamine or amprolium solution was added. Based on the results of the experiments examining the effects of the antimetabolites on glioblastoma cell MTT metabolic activity, final concentrations of 100 µM oxythiamine and 200 µM amprolium were selected for subsequent experiments. At these concentrations, the tested antimetabolites began to exert their maximum inhibitory effects on cell viability. After a further 24 h of incubation, the medium was removed and replaced with fresh medium containing oxythiamine at 100 µM, amprolium at 200 µM, with or without TMZ at concentrations ranging from 10 to 500 µM, either alone or in the relevant combinations. DMSO controls corresponding to the DMSO concentrations present in the 200 and 500 µM TMZ treatments were also included. After 72 h of incubation, an MTT assay was performed. Each treatment condition was tested in duplicate on each plate. At least five independent experiments were conducted for each cell line.

### 2.4. Cell Viability Analysis Using the MTT Assay

Cell viability and the effects of the tested compounds were assessed using the MTT assay [53,54], one of the most commonly used cell viability assays. The MTT solution was prepared by dissolving MTT (475989; Merck) in DPBS (14190-144; Gibco) at a concentration of 5 mg/mL. The solution was subsequently diluted in Medium 199 supplemented with 10% FBS to obtain a final MTT concentration of 0.59 mg/mL. At the end of the incubation period with the tested compounds, the medium was removed and replaced with the MTT solution. The cells were incubated with MTT for 45 min in the case of T98G cells and for 1.5 h in the case of LN-229 cells. The MTT-containing medium was then removed, and 200 µL of DMSO (34869; Merck) was added to each well. After the formazan precipitate had dissolved, absorbance was measured at 570 nm, with 720 nm used as the reference wavelength, using a Synergy LX Multi-Mode Reader (BioTek Instruments, Inc., Winooski, VT, USA).

### 2.5. Effects of Temozolomide and Hypoxia on the Expression of Genes Involved in Vitamin B_1_ Metabolism

To better characterize the regulation of vitamin B_1_ metabolism in glioblastoma, the effects of hypoxia and TMZ on the expression of genes encoding proteins involved in thiamine metabolism were investigated. Solid tumors also contain regions exposed to persistent oxygen deficiency, referred to as chronic hypoxia [55,56]. The effects of chronic hypoxia on the expression of genes involved in vitamin B_1_ metabolism were therefore also analyzed.

LN-229 and T98G cells were seeded in 6-well plates (703001; NEST) at a density of 100,000 cells/well. After 72 h of incubation, the medium was removed and replaced with medium containing TMZ. A TMZ stock solution was prepared at a concentration of 34 mg/mL, corresponding to 175 mM, and was used to obtain final TMZ concentrations of 100 and 500 µM. DMSO was used as the vehicle control. Cells in one well were incubated in Medium 199 supplemented with antibiotics and 10% FBS without TMZ or DMSO. The cells were incubated for 24 h under two different conditions: normoxia and chronic hypoxia. After incubation, the cells were collected for quantitative reverse transcription real-time polymerase chain reaction (qRT-PCR) analysis. Five independent replicates of each experiment were performed for each cell line.

Hypoxic conditions were generated using an incubation chamber of an Agilent BioTek Lionheart FX automated microscope (BioTek Instruments, Inc.). A water-saturated gas mixture containing 1% O_2_, 5% CO_2_, and 94% N_2_ was used. To confirm that hypoxic conditions had been successfully generated, hypoxia-inducible factor-1α (HIF-1α) protein expression was assessed by Western blotting in cells collected from one well. Under chronic hypoxia, the maximum HIF-1α protein level is observed after approximately 4–6 h or 24 h [57,58,59]. Therefore, to verify the chronic-hypoxia conditions, cells were exposed to chronic hypoxia for 6 h or 24 h and subsequently collected for protein isolation and Western blot analysis.

### 2.6. Analysis of Tumor Samples Obtained from Patients with Glioblastoma

The collection and initial analysis of glioblastoma tissue samples were approved by the local bioethics committee (KB-0012/96/14) as part of a study investigating purinergic receptors in glioblastoma. The committee subsequently approved the use of the previously collected samples in studies on chemokines and lipid metabolism (KB-0012/96/14-A-3). More recently, the Bioethics Committee of the Pomeranian Medical University issued an opinion confirming that there were no ethical objections to using these previously collected glioblastoma samples for analyses of vitamin B_1_ metabolism (KB.008,009.2026; date of approval: 13 January 2026). All procedures were conducted in accordance with the Declaration of Helsinki. Written informed consent to participate in the study was obtained from all patients.

Samples were collected from 30 patients with glioblastoma (*n* = 30), including 12 women and 18 men. Because tissue from all three regions was not available for every patient, the number of samples differed between regions. Peritumoral area samples were available from 27 patients (11 women and 16 men), enhancing tumor region samples from 24 patients (12 women and 12 men), and tumor core samples from 27 patients (11 women and 16 men) (Table 1).

Glioblastoma samples were collected from two tumor regions: the non-enhancing tumor core and the enhancing tumor region. This distinction is commonly used in studies of glioblastoma [60,61]. Because the collection of healthy human brain tissue for use as a control raises substantial ethical concerns, tissue from the peritumoral area was used as the reference tissue. The use of peritumoral tissue as a reference is well established in studies of glioblastoma [62].

Glioblastoma tumor samples and peritumoral tissue were collected using the same procedures as described in our previous studies, in which the same tissue samples were analyzed [63,64].

### 2.7. Quantitative Reverse Transcription Real-Time PCR (qRT-PCR)

The analysis focused on the following genes involved in vitamin B_1_ metabolism: *SLC19A2* [11] and *SLC19A3* [12], which encode plasma membrane thiamine transporters; *TPK1* [17], which encodes the enzyme responsible for converting thiamine into its active coenzyme form; and *SLC25A19* [18], which encodes a mitochondrial transporter responsible for transporting TPP into mitochondria. The expression of *SLC35F3* [15], a poorly characterized gene encoding a thiamine transporter, and *SLC44A4*, which encodes a membrane transporter of TPP and acetylcholine [16], was also analyzed.

The expression levels of *SLC19A2*, *SLC19A3*, *TPK1*, *SLC25A19*, *SLC35F3*, and *SLC44A4* were quantitatively assessed using qRT-PCR. Total RNA was isolated from 300,000 cells stored at −80 °C using the RNeasy Mini Kit (74104; Qiagen, Hilden, Germany). Total RNA was isolated from tissue samples using the RNeasy Lipid Tissue Mini Kit (74804; Qiagen). The concentration and purity of the isolated RNA were assessed using a NanoDrop ND-1000 spectrophotometer (NanoDrop Technologies, Wilmington, DE, USA). Reverse transcription was performed using 1 µg of total RNA in a final reaction volume of 20 µL with the Omniscript RT Kit (205113; Qiagen), according to the manufacturer’s instructions. qRT-PCR was performed using a 7500 Fast Real-Time PCR System (Applied Biosystems, Waltham, MA, USA) and Power SYBR Green PCR Master Mix (A25776; Applied Biosystems). The master mix contained AmpliTaq Gold DNA polymerase, deoxynucleotides, SYBR Green dye, and ROX reference dye for fluorescence signal normalization. Amplification was monitored in real time by measuring fluorescence intensity, which was proportional to the amount of PCR product generated. Cycling conditions were determined according to the manufacturer’s instructions, established according to the manufacturer’s recommendations, and optimized for the primers used. All reactions were performed in duplicate, and mean Ct values were used for subsequent calculations. Melting curve analysis was performed after amplification to verify the specificity of the PCR products. Amplification efficiencies were verified and ranged from 90% to 110% for analyzed genes.

Each sample was analyzed in duplicate, and the mean of the two measurements was used in subsequent analyses. The expression level of each target gene was normalized to that of the endogenous reference gene glyceraldehyde-3-phosphate dehydrogenase (*GAPDH*) in analyses of patient tumor samples and experiments examining the effects of TMZ on the expression of genes involved in thiamine metabolism. In experiments examining the effects of hypoxia on the expression of these genes, TATA-box binding protein (*TBP*) was used as the endogenous reference gene because hypoxia increases *GAPDH* expression [65]. Reaction specificity was verified by melting-curve analysis, which confirmed the amplification of a single PCR product. Relative expression levels were calculated using the 2^−ΔCt^ method.

The following primer sequences were used:

*SLC19A2*

Forward: 5′-GGCCGGACAAGAACCTGAC-3′

Reverse: 5′-ACACAGGAAACAGTAGCACCA-3′

*SLC19A3*

Forward: 5′-TCCCCGTTTGGACATACTCCT-3′

Reverse: 5′-GCTTGTAGCGGACATAATCGGT-3′

*TPK1*

Forward: 5′-AGCCTTTGGACAACTATTTTCGT-3′

Reverse: 5′-AGCTCACATCCCTTAGTAGCA-3′

*SLC25A19*

Forward: 5′-GGTCTGTGTCTGGACTTGTTAC-3′

Reverse: 5′-GCTGAAGCTGGAAACGGATCT-3′

*SLC35F3*

Forward: 5′-GGGATTCGAGAGTTTCCCAGC-3′

Reverse: 5′-GATCCTCCTTAATCGCCTCGT-3′

*SLC44A4*

Forward: 5′-ATGCCCGAGACATCAGTGTTA-3′

Reverse: 5′-AACAGTAGGCTCAAGACCAGA-3′

*TBP*

Forward: 5′- CCACTCACAGACTCTCACAAC-3′

Reverse: 5′-CTGCGGTACAATCCCAGAACT-3′

### 2.8. Western Blot Analysis

To confirm that hypoxic conditions had been successfully generated, HIF-1α protein levels were assessed by Western blotting. Under hypoxic conditions, HIF-1α does not undergo rapid proteolytic degradation, resulting in its intracellular accumulation [58,59]. The accumulated protein subsequently induces the expression of hypoxia-responsive genes. Under normoxic conditions, HIF-1α is rapidly degraded. Under chronic hypoxia, the maximum accumulation of HIF-1α protein occurs after approximately 4–6 h [58,59]. Western blot analysis was therefore performed after 6 h of chronic hypoxia. HIF-1α protein levels were also assessed after 24 h of chronic hypoxia, corresponding to the time point at which gene expression was analyzed by qRT-PCR.

After incubation under hypoxic conditions, the cells were washed three times with DPBS containing calcium and magnesium ions (CC711-0500; GeneDireX, Taoyuan City, Taiwan). Proteins were extracted by incubating the cells for 20 min at 4 °C in radioimmunoprecipitation assay buffer (RIPA) (R0278; Merck) supplemented with protease inhibitors (4906845001 and 11836170001; Roche, Basel, Switzerland). The samples were then centrifuged at 13,000× *g* for 20 min at 4 °C, and the supernatants were collected and frozen until Western blot analysis.

Protein concentrations were determined using the Pierce BCA Protein Assay Kit (23225; Thermo Fisher Scientific, Waltham, MA, USA) to ensure equal protein loading. Proteins were separated by sodium dodecyl sulfate-polyacrylamide gel electrophoresis (SDS-PAGE) using a Mini-PROTEAN Tetra Cell system (Bio-Rad) and 4–16% gradient resolving gels (M00660; GenScript, Piscataway, NJ, USA). A total of 10 µg of protein was loaded per lane. Proteins were transferred using a Tetra Blotting Module (Bio-Rad) onto 0.45 µm polyvinylidene difluoride membranes (PVDF) (88518; Thermo Fisher Scientific). After transfer, the membranes were blocked with 5% non-fat dry milk for 1 h at room temperature. Primary and secondary antibodies were also diluted in this blocking solution. The membranes were incubated overnight with the primary antibodies. After three washes with Tris-buffered saline (TBS) (ZXB-06-199; ZELLX^®^, Berlin, Germany) with 0.1% Tween 20 (P1379; Merck), the membranes were incubated with the appropriate secondary antibodies for 45 min.

The following primary antibodies were used:

- Rabbit polyclonal anti-HIF-1α antibody (20960-1-AP; Proteintech, Rosemont, IL, USA);
- Rabbit monoclonal anti-GAPDH antibody (ab8245; Abcam, Cambridge, UK);
- Mouse monoclonal anti-α-tubulin antibody (ab7291; Abcam).

The following horseradish peroxidase-conjugated secondary antibodies were used:

- Goat anti-rabbit IgG HRP H&L antibody (ab97051; Abcam);
- Goat anti-mouse IgG HRP H&L antibody (ab6789; Abcam).

Protein signals were detected using the ECL Select Western Blotting Detection Reagent (RPN2235; GE Healthcare, Chicago, IL, USA) and visualized using a Molecular Imager ChemiDoc XRS+ system (Bio-Rad).

**Figure 1:**
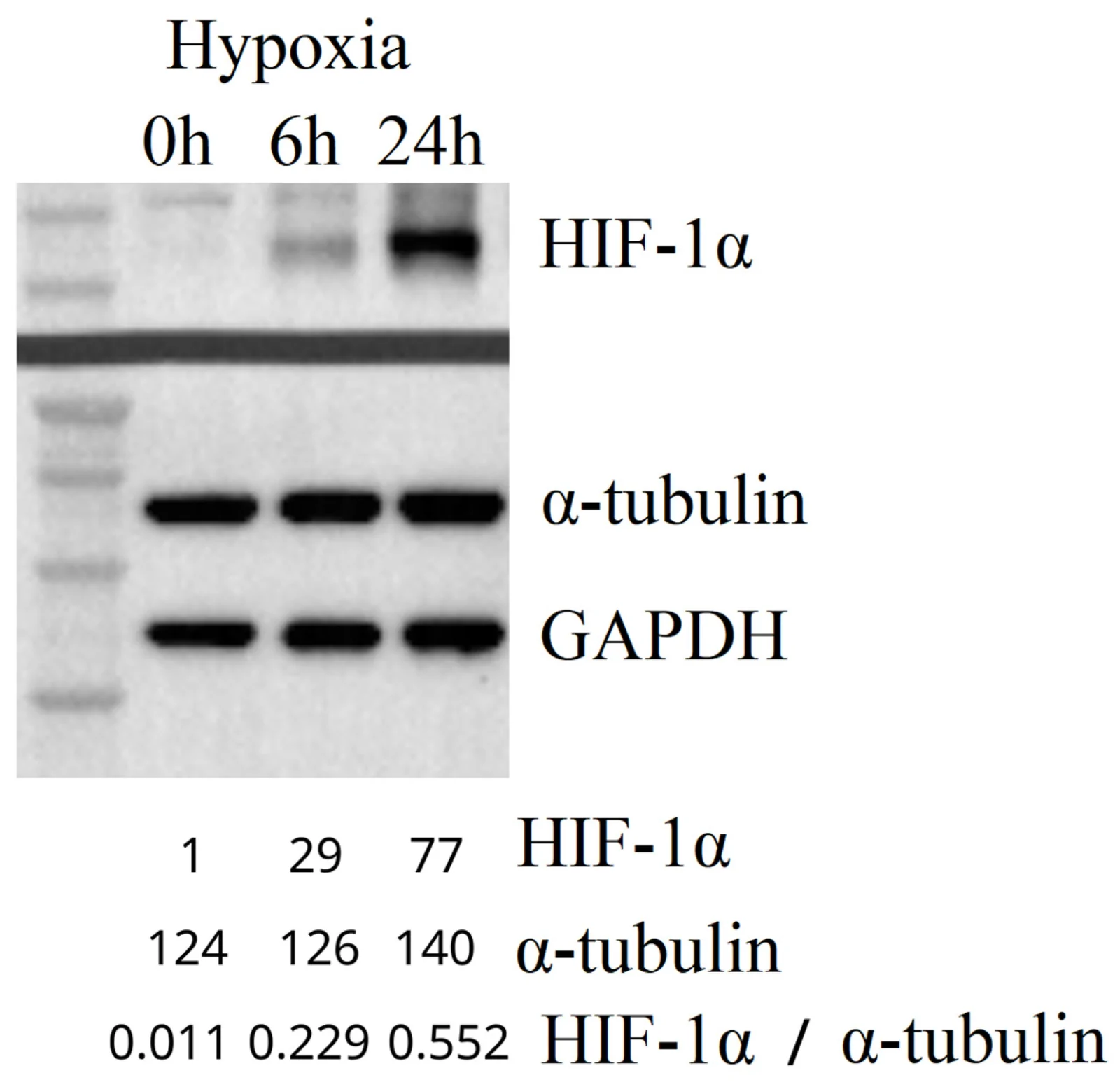
Western blot analysis of HIF-1α protein levels under chronic hypoxic conditions. The whole blots (uncropped blots) are shown in the Supplementary File.

The Western blot results are presented below (Figure 1). Densitometric analysis was performed using ImageJ version 1.54g (National Institutes of Health (NIH), Bethesda, MD, USA) [66,67]. The analysis showed that chronic hypoxia increased HIF-1α protein levels, confirming the successful establishment of chronic hypoxic conditions.

### 2.9. Immunohistochemical Staining

Tissue preparation and immunohistochemical procedures were performed as described in our previous publications [68,69]. Tissue sections were incubated with a primary polyclonal rabbit antibody against TPK1 (10942-1-AP; Proteintech). Negative controls were prepared by omitting the primary antibody. IHC-stained slides were scanned using a 3DHISTECH Pannoramic MIDI II scanner (Sysmex Polska Sp. z o.o., Warsaw, Poland) at 20× magnification. Digital image analysis was performed using Pattern Quant software v.2.3 (3DHISTECH Kft, Budapest, Hungary).

### 2.10. Statistical Analysis

Statistical analyses were performed using Statistica v.13.1 for Windows (StatSoft, Inc., Krakow, Poland). The normality of data distributions was assessed using the Shapiro–Wilk test (α = 0.05). Because the data were not normally distributed, nonparametric tests were used. The effects of TMZ, thiamine, oxythiamine, and amprolium on the metabolic activity of LN-229 and T98G cells, as measured by the MTT assay, were analyzed using the Wilcoxon signed-rank test. Differences in the effects of vitamin B_1_ antimetabolites across treatment durations were assessed using the Mann–Whitney U test. This test was also used to compare the effects of TMZ alone with those of TMZ in combination with vitamin B_1_ antimetabolites.

The expression levels of six genes involved in vitamin B_1_ metabolism were analyzed in glioblastoma samples from 30 patients (12 women and 18 men). Analyses were performed for the entire study population and separately for women and men, considering three tissue regions: the peritumoral area, the enhancing tumor region, and the tumor core. Because the data were not normally distributed, differences among tissue regions were assessed using the Friedman test (α = 0.05), followed by pairwise comparisons using the Wilcoxon signed-rank test with Bonferroni correction.

Associations among the expression levels of the analyzed genes were assessed using Spearman’s rank correlation coefficient (*r_S_*). The resulting *p*-values were adjusted for multiple testing using the Benjamini–Hochberg procedure to control the false discovery rate (FDR). Adjusted *p*-values ≤ 0.05 were considered statistically significant.

Age at surgery, body weight, and height were summarized for the entire study population and separately for women and men. Body mass index (BMI; kg/m^2^) was calculated from body weight and height. The normality of the distributions of these variables was assessed using the Shapiro–Wilk test.

Spearman’s rank correlation coefficient (*r_S_*; α = 0.05) was used to assess associations between the expression levels of the six genes in the three tissue regions and patient characteristics, including age, body weight, height, and BMI.

## 3. Results

### 3.1. Thiamine Promotes Glioblastoma Cell Viability but Does Not Protect Cells Against TMZ

**Figure 2:**
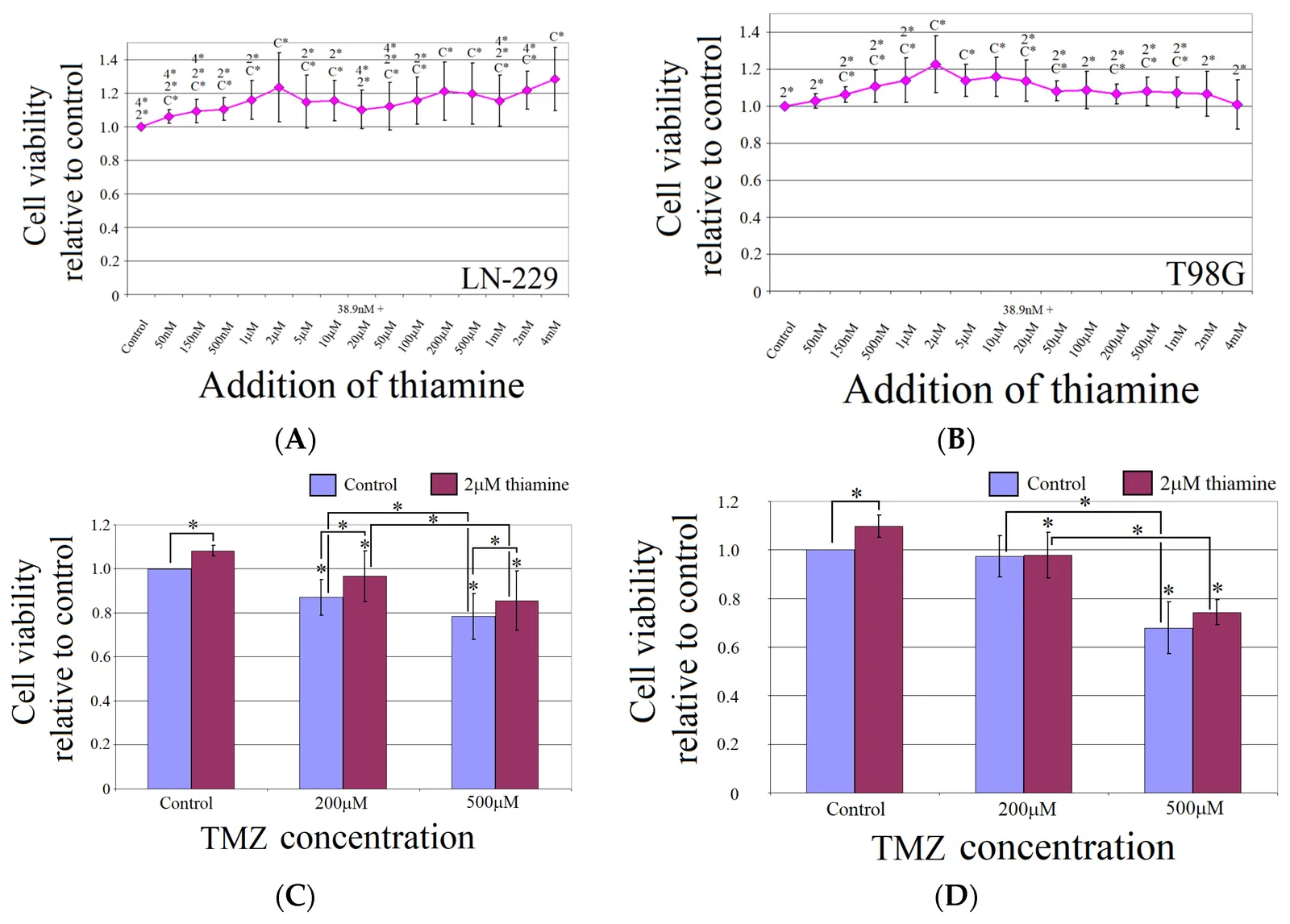
Effect of thiamine on the viability of LN-229 and T98G cells. The effects of thiamine on (**A**) LN-229 and (**B**) T98G cells. The combined effects of TMZ and 2 µM of thiamine were analyzed in (**C**) LN-229 and (**D**) T98G cells. Before the experiment, the cells were cultured for at least one week in Medium 199 supplemented with 10% FBS. This medium contains a near-physiological concentration of thiamine and was also used throughout the experiment. Cells were seeded in 96-well plates at a density of 700 cells/well and cultured with different thiamine concentrations. The medium was replaced after three days. After a total of six days of incubation, the medium was removed, and an MTT assay was performed. C* and * Significant difference compared with the control, *p* < 0.05; 2*, significant difference compared with cell viability at 2 µM thiamine, *p* < 0.05; 4*, significant difference compared with cell viability at 4 mM thiamine, *p* < 0.05. Baseline thiamine concentration: 38.9 nM.

To determine the effect of thiamine on glioblastoma cell viability, LN-229 and T98G cells were cultured with different concentrations of vitamin B_1_. After six days of incubation, thiamine increased the MTT metabolic activity of both cell lines. This effect tended to increase up to a thiamine concentration of 2 µM. At this concentration, cell MTT metabolic activity reached 1.23 ± 0.15-fold and 1.23 ± 0.21-fold relative to the control in T98G and LN-229 cells, respectively. At higher concentrations, the viability-promoting effect of thiamine gradually declined toward the control level. However, millimolar thiamine concentrations continued to stimulate LN-229 cell viability. At 4 mM thiamine, LN-229 cell viability was 1.29 ± 0.19-fold relative to control (Figure 2). These findings indicate that thiamine promotes the viability of glioblastoma cells. MTT signal was observed at a thiamine concentration higher than the estimated physiological concentration.

To determine whether increased thiamine availability affected the response to chemotherapy with TMZ, the cells were initially cultured in medium supplemented with thiamine for two days and were then incubated with TMZ in the continued presence of thiamine. A thiamine concentration of 2 µM was used because this concentration produced the strongest increase in cell viability in the preceding experiment.

During the five-day incubation period, thiamine alone increased the viability of LN-229 and T98G cells by 8.1 ± 2.4% and 9.6 ± 4.5%, respectively, compared with cells cultured without additional thiamine (*p* = 0.028). TMZ reduced LN-229 cell viability at concentrations of 200 and 500 µM and T98G cell viability at 500 µM (*p* = 0.028). Overall, thiamine did not substantially alter the response to TMZ, which reduced cell viability in a concentration-dependent manner both in the presence and absence of additional thiamine.

In LN-229 cells, 200 µM TMZ reduced viability to 87.7 ± 8.1% without additional thiamine and to 89.2 ± 9.8% in the presence of thiamine. At 500 µM TMZ, the corresponding values were also similar: 78.3 ± 10.4% without thiamine and 79.0 ± 11.8% with thiamine. Nevertheless, LN-229 cells treated with both TMZ and thiamine exhibited higher viability than cells treated with TMZ alone (*p* = 0.028).

In T98G cells, 200 µM TMZ alone did not significantly affect viability, which remained at 97.2 ± 8.5% relative to the control (*p* = 0.92). However, when 200 µM TMZ was added to thiamine-treated T98G cells, viability decreased from 109.6 ± 4.5% to 97.7 ± 9.4% (*p* = 0.028). Thus, under these conditions, TMZ counteracted the viability-promoting effect of thiamine.

### 3.2. Vitamin B_1_ Antimetabolites Reduce the Viability of Glioblastoma Cells

To investigate the dependence of glioblastoma cells on thiamine metabolism, two vitamin B_1_ antimetabolites, oxythiamine and amprolium, were used. LN-229 and T98G cells were incubated with the compounds in a medium containing a low, near-physiological concentration of thiamine.

After 72 h of incubation, oxythiamine significantly reduced cell viability at concentrations of 5 µM and above in LN-229 cells and 10 µM and above in T98G cells. In T98G cells, viability decreased to 67 ± 5% at an oxythiamine concentration of 50 µM and remained relatively unchanged at higher concentrations. At the highest concentration tested, 1 mM, oxythiamine produced a further reduction in T98G cell MTT metabolic activity to 56 ± 9%.

**Figure 3:**
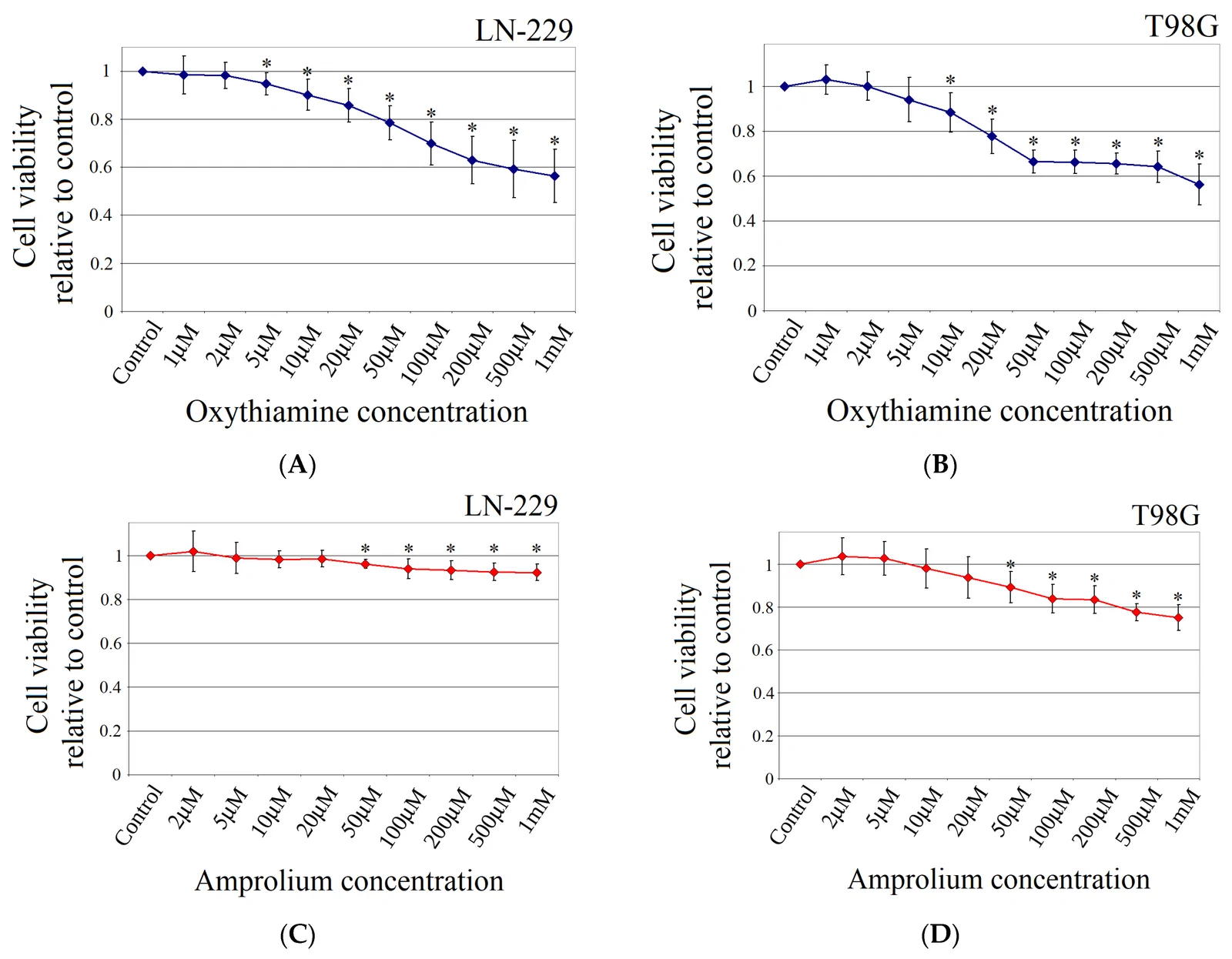

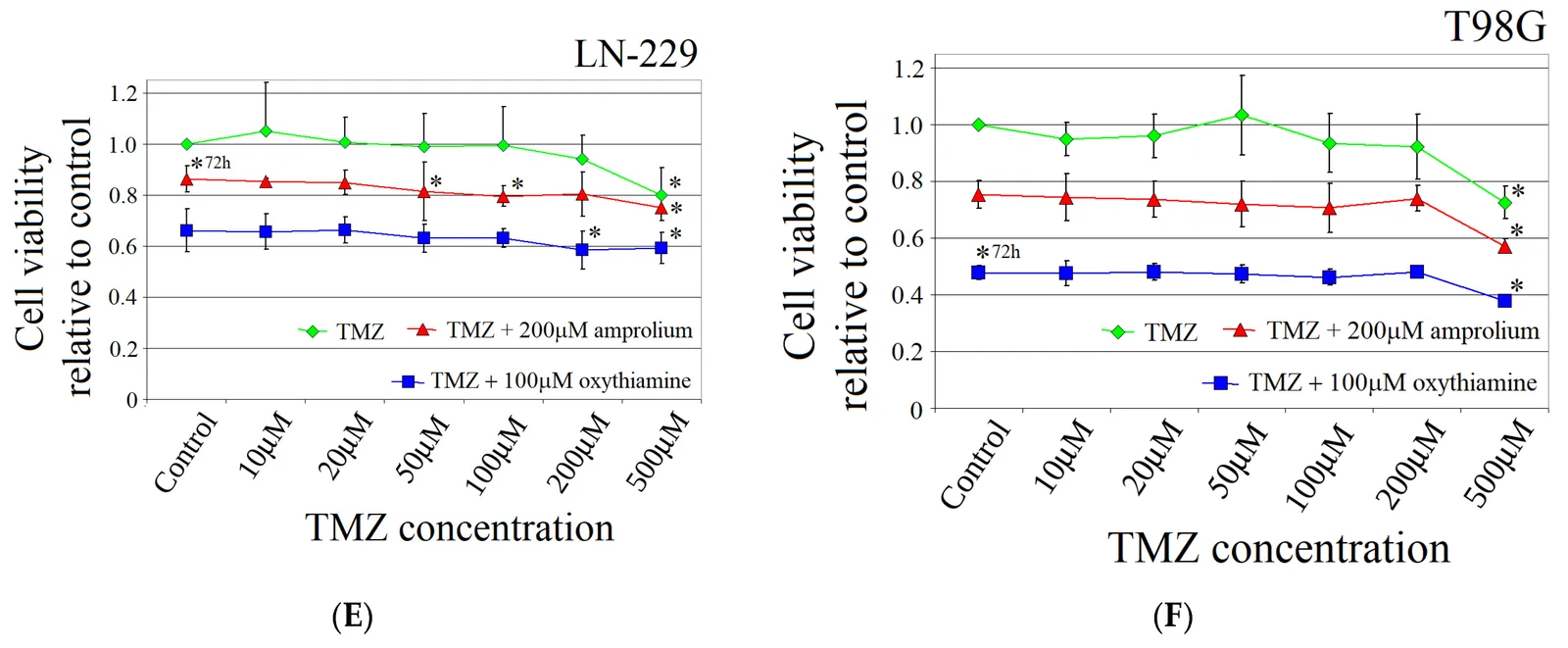
Effects of oxythiamine and amprolium on the viability of glioblastoma cells and their combined effects with TMZ. The effects of oxythiamine on (**A**) LN-229 and (**B**) T98G cells and the effects of amprolium on (**C**) LN-229 and (**D**) T98G cells are shown. The combined effects of TMZ and the tested thiamine antimetabolites were analyzed in (**E**) LN-229 and (**F**) T98G cells. Before the experiment, the cells were cultured for at least one week in Medium 199 supplemented with 10% FBS. This medium contains a near-physiological concentration of thiamine and was used throughout the experiment. Cells were seeded in 96-well plates at a density of 2000 cells/well. After 48 h, the medium was replaced with medium containing the specified concentration of a thiamine antimetabolite. After a further 72 h of incubation, an MTT assay was performed. In the experiments examining the combined effects of the antimetabolites and TMZ, the cells were additionally preincubated with the antimetabolites for 24 h. The results were expressed relative to untreated control cells. * Significant difference compared with the control, *p* < 0.05; *72, significant difference compared with treatment with the respective compound without preincubation, *p* < 0.05.

In LN-229 cells, oxythiamine significantly reduced cell viability at concentrations of 5 µM and above. The magnitude of this effect increased with the concentration of the antimetabolite, and MTT metabolic activity reached 56 ± 11% at the highest oxythiamine concentration tested. Thus, the viability of the two cell lines was similar at the highest oxythiamine concentrations (Figure 3).

Amprolium significantly reduced cell viability at concentrations of 50 µM and above in both cell lines. Increasing amprolium concentrations were associated with progressively lower cell viability. In LN-229 cells, MTT metabolic activity remained at approximately 93% of the control value over the concentration range from 100 µM to 1 mM, with no significant differences among concentrations within this range. In T98G cells, viability decreased in a concentration-dependent manner, reaching 75 ± 6% of the control value at the highest concentration tested, 1 mM. These findings indicate that glioblastoma cell MTT metabolic activity is dependent on thiamine metabolism only to a limited extent.

To assess the therapeutic potential of vitamin B_1_ antimetabolites in glioblastoma treatment, we investigated whether these compounds enhanced the effects of TMZ. At the highest concentration tested, 500 µM, TMZ reduced LN-229 and T98G cell viability to 80 ± 11% and 73 ± 6%, respectively. Thus, the effects of TMZ were comparable between the two cell lines.

Based on the concentration-response curves, 100 µM oxythiamine and 200 µM amprolium were selected for the combination experiments because these concentrations were close to the onset of the maximum inhibitory effects observed for the respective compounds. Under the conditions used in these experiments, 100 µM oxythiamine reduced T98G and LN-229 cell viability to 48 ± 2% and 66 ± 8%, respectively. Amprolium at 200 µM reduced T98G and LN-229 cell viability to 76 ± 5% and 86 ± 3%, respectively. In LN-229 cells, preincubation with 200 µM amprolium significantly reduced viability compared with incubation with amprolium without preincubation (86 ± 3% vs. 93 ± 4%; *p* = 0.022). A similar effect was observed in T98G cells treated with oxythiamine (48 ± 2% vs. 66 ± 5%; *p* = 0.00117). These viability values were lower than those obtained in the preceding experiments because the total period of exposure to the antimetabolites was 24 h longer.

The delayed effects may result from inhibition of TPP-dependent enzymes involved in the production of metabolites required by cancer cells. Inhibition of these enzymes may initially reduce metabolite synthesis, whereas the intracellular pools of the affected metabolites become depleted only after prolonged exposure. Cells were therefore preincubated with the antimetabolites for 24 h before subsequent incubation with the antimetabolites and TMZ.

In T98G cells treated with the vitamin B_1_ antimetabolites and TMZ, viability was 78 ± 5%, compared with 73 ± 6% in cells treated with TMZ alone. Viability was therefore significantly higher in the combination-treatment group (*p* = 0.027). A similar pattern was observed in LN-229 cells: treatment with TMZ and vitamin B_1_ antimetabolites reduced viability to 88 ± 5%, whereas TMZ alone reduced viability to 80 ± 11%. However, this difference was not statistically significant (*p* = 0.083). These findings show that vitamin B_1_ antimetabolites did not enhance the effects of TMZ and may even have reduced its activity under the experimental conditions used.

### 3.3. TMZ Does Not Appear to Directly Affect Vitamin B_1_ Metabolism in Glioblastoma Cells

In the final in vitro experiment, we investigated whether TMZ and chronic hypoxia affect vitamin B_1_ metabolism in glioblastoma cells. This experimental model was designed to assess the effects of chemotherapy and the tumor microenvironment on the processes associated with vitamin B_1_ metabolism. The following genes were analyzed: *TPK1*, *SLC19A2*, *SLC19A3*, *SLC25A19*, *SLC35F3*, and *SLC44A4*.

No detectable mRNA expression of *SLC35F3* or *SLC44A4* was observed in LN-229 cells, whereas *SLC35F3* mRNA expression was not detectable in T98G cells.

**Figure 4:**
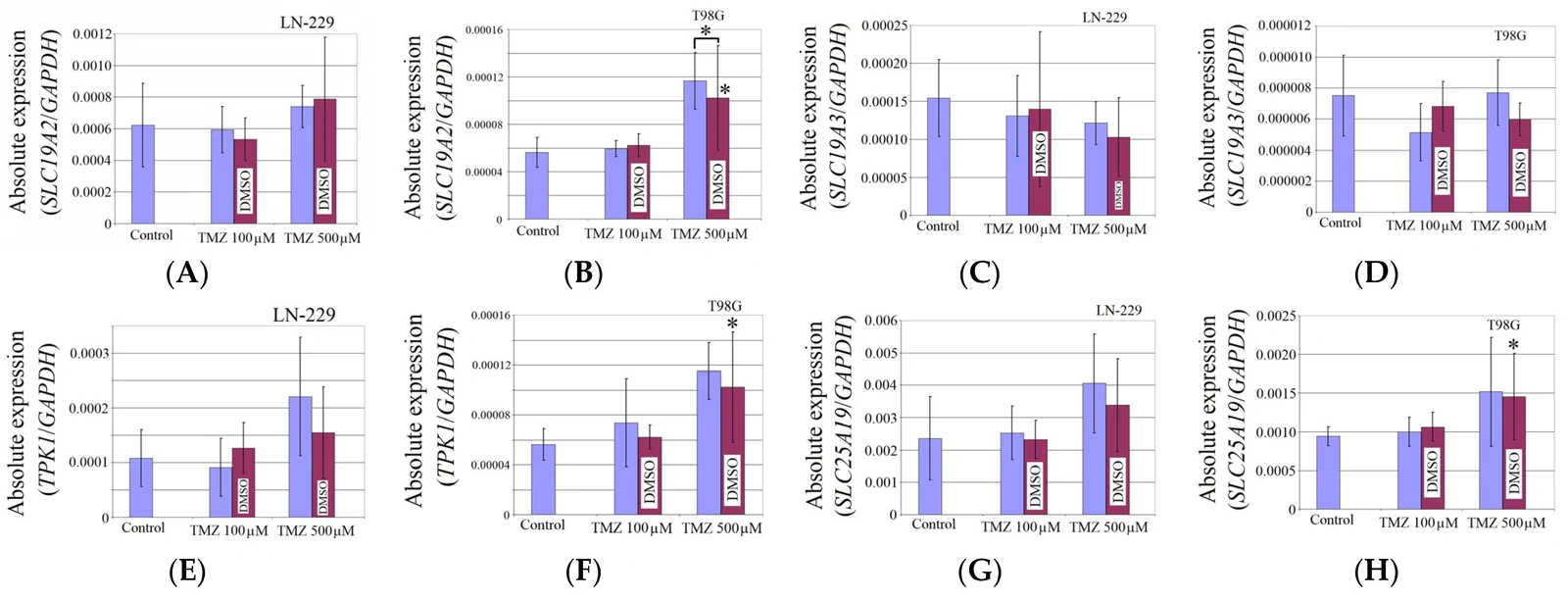

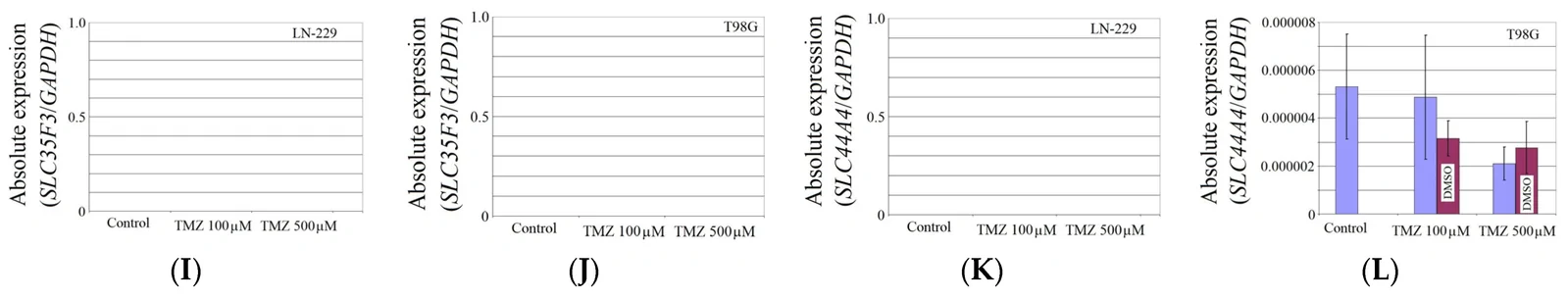
Effects of TMZ on vitamin B_1_ metabolism. The effects of TMZ on expression levels of *SLC19A2* (**A**,**B**), *SLC19A3* (**C**,**D**), *TPK1* (**E**,**F**), *SLC25A19* (**G**,**H**), *SLC35F3* (**I**,**J**), and *SLC44A4* (**K**,**L**) in LN-229 (**A**,**C**,**E**,**G**,**I**,**K**) and T98G cells (**B**,**D**,**F**,**H**,**J**,**L**). Before the experiment, the cells were cultured for at least one week in Medium 199 supplemented with 10% FBS. This medium contains a near-physiological concentration of thiamine and was used throughout the experiment. Cells were seeded in 6-well plates at a density of 100,000 cells/well. After 72 h, the medium was removed and replaced with medium containing TMZ. The cells were incubated for 24 h. After incubation, the cells were collected for qRT-PCR analysis. * Significant difference compared with the control, *p* < 0.05.

The effect of TMZ on vitamin B_1_ metabolism could not be clearly distinguished from the effect of DMSO (Figure 4). In T98G cells, DMSO alone significantly increased the expression of *TPK1* (*p* = 0.0079), *SLC19A2* (*p* = 0.0079), and *SLC25A19* (*p* = 0.0079). However, treatment with TMZ dissolved in DMSO further increased *SLC19A2* expression compared with DMSO alone (*p* = 0.0079). These findings make it difficult to determine the specific effect of TMZ independently of the solvent. TMZ is poorly soluble in aqueous solutions and is therefore commonly dissolved in DMSO before being added to experimental cultures. However, because the solubility of TMZ in DMSO is limited, the amount of DMSO introduced together with TMZ may itself influence gene expression and thus confound interpretation of the results.

**Figure 5:**
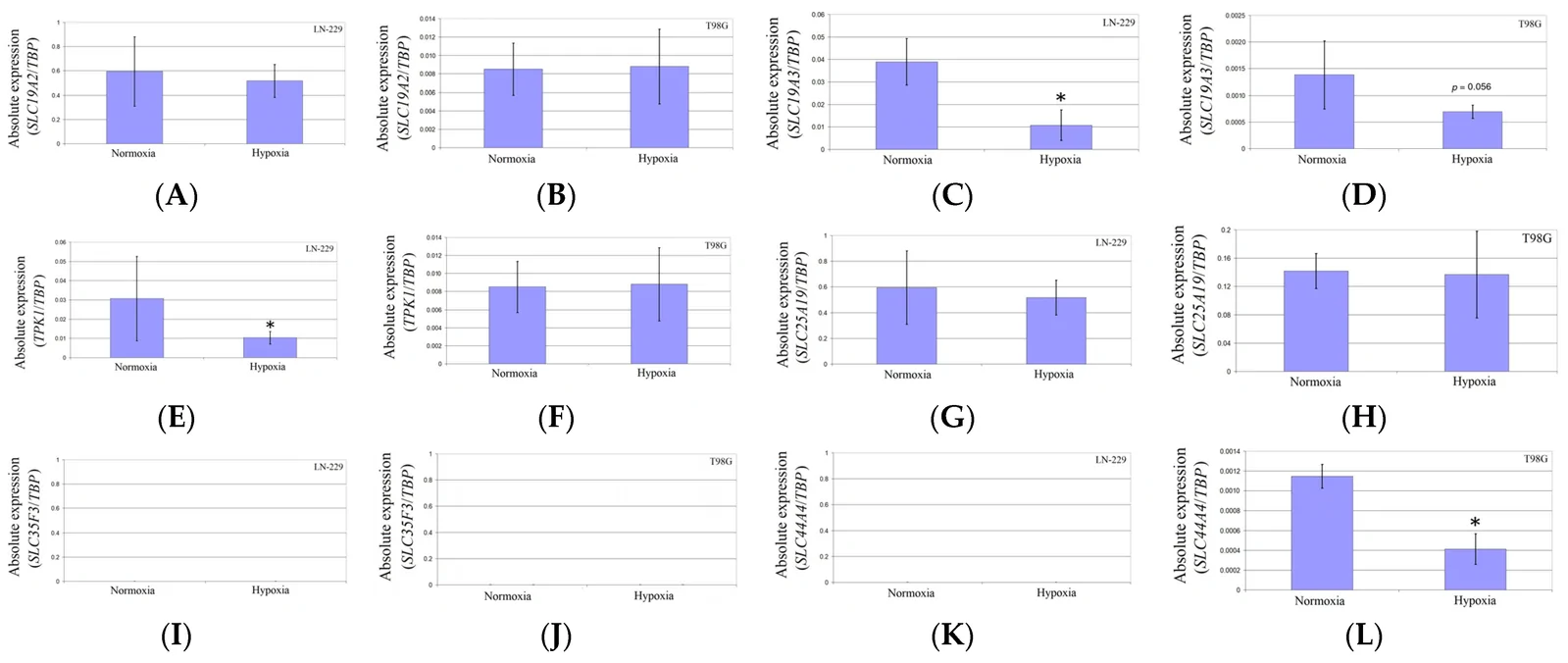
Effects of hypoxia on vitamin B_1_ metabolism. The effects of hypoxia on expression levels of *SLC19A2* (**A**,**B**), *SLC19A3* (**C**,**D**), *TPK1* (**E**,**F**), *SLC25A19* (**G**,**H**), *SLC35F3* (**I**,**J**), and *SLC44A4* (**K**,**L**) in LN-229 (**A**,**C**,**E**,**G**,**I**,**K**) and T98G cells (**B**,**D**,**F**,**H**,**J**,**L**). Before the experiment, the cells were cultured for at least one week in Medium 199 supplemented with 10% FBS. This medium contains a near-physiological concentration of thiamine and was used throughout the experiment. Cells were seeded in 6-well plates at a density of 100,000 cells/well. After 72 h, the medium was replaced. The cells were incubated for 24 h under two different conditions: normoxia and chronic hypoxia. Hypoxic conditions were generated using a water-saturated gas mixture containing 1% O_2_, 5% CO_2_, and 94% N_2_. After incubation, the cells were collected for qRT-PCR analysis. * Significant difference compared with the control, *p* < 0.05.

Chronic hypoxia altered the expression of genes involved in vitamin B_1_ metabolism in glioblastoma cells (Figure 5), although the pattern of changes differed between the two cell lines. In LN-229 cells, chronic hypoxia significantly decreased *SLC19A3* expression (*p* = 0.016) and *TPK1* (*p* = 0.016). In T98G cells, chronic hypoxia significantly decreased the expression of *SLC44A4* (*p* = 0.0079).

### 3.4. Vitamin B_1_ Metabolism Is Less Active in Glioblastoma Tissue than in the Peritumoral Area

In the next stage of the study, glioblastoma tissue samples were analyzed to determine the expression levels of genes involved in vitamin B_1_ metabolism. The results were compared with those obtained for the peritumoral area, which is an appropriate reference tissue for studies of brain tumors [62]. The expression of *SLC19A2*, *SLC19A3*, *TPK1*, *SLC25A19*, *SLC35F3*, and *SLC44A4* was analyzed.

The statistical significance of differences was assessed using the Friedman test (α = 0.05), a nonparametric test for related samples. Pairwise comparisons were performed using the Wilcoxon signed-rank test (α = 0.05). Bonferroni correction was applied to control the risk of type I error associated with multiple comparisons. Accordingly, a difference between a given pair of tissue regions (e.g., the peritumoral area vs. the enhancing tumor region) was considered statistically significant at *p* < 0.0167.

**Figure 6:**
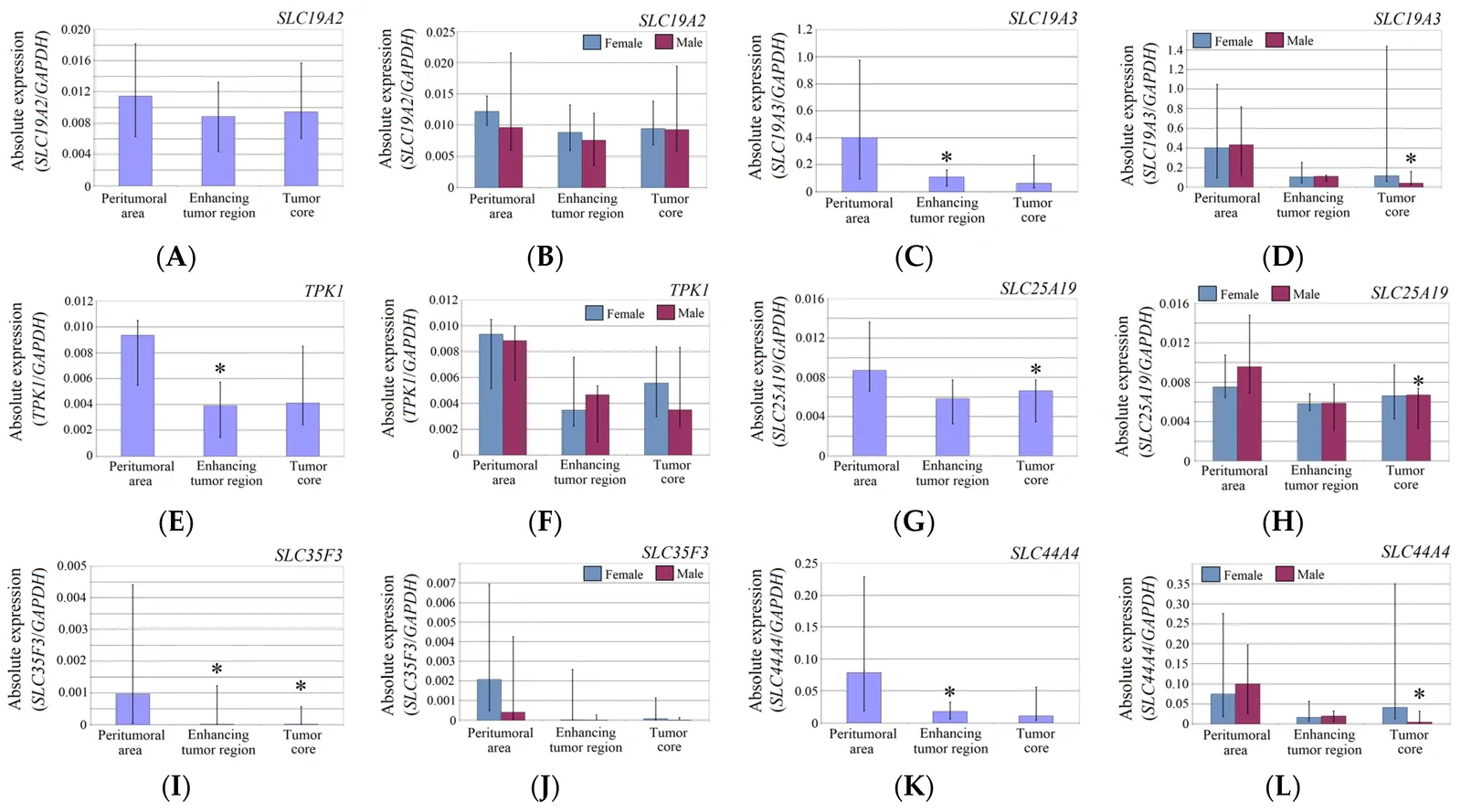
Expression levels of genes involved in vitamin B_1_ metabolism in glioblastoma tissue. Expression levels of *SLC19A2* (**A**,**B**), *SLC19A3* (**C**,**D**), *TPK1* (**E**,**F**), *SLC25A19* (**G**,**H**), *SLC35F3* (**I**,**J**), and *SLC44A4* (**K**,**L**) are presented for all patients combined (**A**,**C**,**E**,**G**,**I**,**K**) and separately according to sex (**B**,**D**,**F**,**H**,**I**,**L**). Tumor samples were collected from 30 patients, including 12 women and 18 men. After RNA isolation, the expression of the selected genes was analyzed by qRT-PCR. Differences among the tissue regions were evaluated using Dunn’s post hoc test; * Significant difference between the respective tumor region and the peritumoral area, *p* < 0.0167.

Gene expression levels differed significantly among the tissue regions examined. The Friedman test revealed statistically significant differences in the expression of five of the six analyzed genes among the peritumoral area, enhancing tumor region, and tumor core. The exception was *SLC19A2*, for which no statistically significant differences were detected among the regions (χ^2^ = 4.3333, *p* = 0.1146); its expression levels were similar across all three regions (Figure 6).

SLC19A3 is one of the principal plasma membrane thiamine transporters [13]. The Friedman test revealed significant differences in *SLC19A3* expression among the tissue regions (χ^2^ = 10.750, *p* = 0.0046). Pairwise comparisons using the Wilcoxon signed-rank test, with a Bonferroni-adjusted significance threshold of *p* < 0.0167, showed a statistically significant difference between the peritumoral area and the enhancing tumor region (*Z* = 2.5714, *p* = 0.0101). No statistically significant differences were found between the peritumoral area and the tumor core (*Z* = 2.2584, *p* = 0.0239) or between the enhancing tumor region and the tumor core (*Z* = 0.6571, *p* = 0.5110).

The next gene analyzed was *TPK1*, which encodes the enzyme responsible for converting thiamine into its active cofactor form [17] and therefore plays a central role in vitamin B1 metabolism. The Friedman test confirmed significant differences in *TPK1* expression among the tissue regions (χ^2^ = 10.2526, *p* = 0.0059). The Wilcoxon signed-rank test, with a Bonferroni-adjusted significance threshold of *p* < 0.0167, showed a statistically significant difference between the peritumoral area and the enhancing tumor region (*Z* = 2.6157, *p* = 0.0089). No statistically significant differences were found between the peritumoral area and the tumor core (*Z* = 2.2584, *p* = 0.0239) or between the enhancing tumor region and the tumor core (*Z* = 1.0286, *p* = 0.3037).

The mitochondrial transporter gene *SLC25A19* was also analyzed [18]. The Friedman test revealed significant differences in *SLC25A19* expression among the tissue regions (χ^2^ = 11.0833, *p* = 0.0039). The Wilcoxon signed-rank test, with a Bonferroni-adjusted significance threshold of *p* < 0.0167, showed a statistically significant difference between the peritumoral area and the tumor core (*Z* = 3.4596, *p* = 0.0005). No statistically significant differences were found between the peritumoral area and the enhancing tumor region (*Z* = 2.0000, *p* = 0.0455) or between the enhancing tumor region and the tumor core (*Z* = 0.4286, *p* = 0.6682).

Two less extensively characterized transporter genes involved in vitamin B_1_ metabolism were also investigated. *SLC35F3* encodes a thiamine transporter [15], whereas *SLC44A4* encodes a transporter of TPP and acetylcholine [16,70].

The Friedman test revealed significant differences in *SLC35F3* expression among the tissue regions (χ^2^ = 8.0889, *p* = 0.0175). The Wilcoxon signed-rank test, with a Bonferroni-adjusted significance threshold of *p* < 0.0167, identified statistically significant differences between the peritumoral area and the enhancing tumor region (*Z* = 2.7143, *p* = 0.0066) and between the peritumoral area and the tumor core (*Z* = 2.9463, *p* = 0.0032). No statistically significant difference was found between the enhancing tumor region and the tumor core (*Z* = 0.0373, *p* = 0.9702).

The Friedman test revealed significant differences in *SLC44A4* expression among the tissue regions (χ^2^ = 9.2500, *p* = 0.0098). The Wilcoxon signed-rank test, with a Bonferroni-adjusted significance threshold of *p* < 0.0167, confirmed a statistically significant difference between the peritumoral area and the enhancing tumor region (*Z* = 2.6286, *p* = 0.0086). No statistically significant differences were found between the peritumoral area and the tumor core (*Z* = 2.2343, *p* = 0.0255) or between the enhancing tumor region and the tumor core (*Z* = 0.9429, *p* = 0.3458).

Overall, these expression patterns suggest that vitamin B_1_ transport and metabolism are less active in glioblastoma tissue than in the peritumoral area. However, the reduced expression of these genes does not exclude their potential involvement in glioblastoma biology.

The tumor samples were also analyzed separately according to patient sex. Among women, no statistically significant differences in the expression of the analyzed genes were observed among the peritumoral area, enhancing tumor region, and tumor core. The Kruskal–Wallis test results were as follows: *SLC19A3* (*H* = 3.3034, *p* = 0.1917), *TPK1* (*H* = 3.3374, *p* = 0.1885), *SLC19A2* (*H* = 1.2472, *p* = 0.5360), *SLC44A4* (*H* = 3.3034, *p* = 0.1917), *SLC35F3* (*H* = 3.6795, *p* = 0.1589), and *SLC25A19* (*H* = 3.8387, *p* = 0.1467). Among men, significant differences were identified only for *SLC19A3* (*H* = 6.7766, *p* = 0.0338) and *SLC44A4* (*H* = 7.1297, *p* = 0.0283). In male patients, *SLC19A3* expression was significantly lower in the tumor core than in the peritumoral area (*p* = 0.0354), whereas the difference between the enhancing tumor region and peritumoral area was not statistically significant (*p* = 0.240). Similarly, *SLC44A4* expression was significantly lower in the tumor core than in the peritumoral area (*p* = 0.0302). The Kruskal–Wallis test results for the remaining genes in male patients were as follows: *TPK1* (*H* = 5.2486, *p* = 0.0725), *SLC19A2* (*H* = 0.9151, *p* = 0.6328), *SLC35F3* (*H* = 5.9338, *p* = 0.0515), and *SLC25A19* (*H* = 5.4468, *p* = 0.0657).

In male patients, statistically significant pairwise differences were confirmed for only three genes: *SLC19A3* (Friedman test: χ^2^ = 8.0000, *p* = 0.0183), *SLC44A4* (χ^2^ = 8.1667, *p* = 0.0169), and *SLC25A19* (χ^2^ = 8.0000, *p* = 0.0183). These differences were found only between the peritumoral area and the tumor core.

The Wilcoxon signed-rank test, with a Bonferroni-adjusted significance threshold of *p* < 0.0167, revealed a statistically significant difference in *SLC19A3* expression between the peritumoral area and the tumor core (*T* = 16.0000, *p* = 0.0072). No statistically significant differences were found between the peritumoral area and the enhancing tumor region (*T* = 10.0000, *p* = 0.0229) or between the enhancing tumor region and the tumor core (*T* = 36.0000, *p* = 0.8139).

The Wilcoxon signed-rank test, with the same adjusted significance threshold, also revealed a statistically significant difference in *SLC44A4* expression between the peritumoral area and the tumor core (*T* = 14.0000, *p* = 0.0052). No statistically significant differences were found between the peritumoral area and the enhancing tumor region (*T* = 10.0000, *p* = 0.0229) or between the enhancing tumor region and the tumor core (*T* = 36.0000, *p* = 0.8139).

Similarly, the Wilcoxon signed-rank test, with the same adjusted significance threshold, revealed a statistically significant difference in *SLC25A19* expression between the peritumoral area and the tumor core (*T* = 10.0000, *p* = 0.0027). No statistically significant differences were found between the peritumoral area and the enhancing tumor region (*T* = 16.0000, *p* = 0.0712) or between the enhancing tumor region and the tumor core (*T* = 39.0000, *p* = 1.0000).

For the remaining genes, the Friedman test results were as follows: *TPK1* (χ^2^ = 8.1667, *p* = 0.01685), *SLC19A2* (χ^2^ = 1.1667, *p* = 0.55804), and *SLC35F3* (χ^2^ = 7.3478, *p* = 0.02538). Although the Friedman test indicated statistically significant differences among tissue regions for *TPK1* and *SLC35F3*, subsequent Wilcoxon signed-rank tests did not identify any statistically significant pairwise differences after Bonferroni correction.

In female patients, no statistically significant differences in gene expression were detected among the tissue regions examined. The Friedman test results were as follows: *SLC19A3* (χ^2^ = 5.6364, *p* = 0.0597), *TPK1* (χ^2^ = 2.2791, *p* = 0.3200), *SLC19A2* (χ^2^ = 2.3636, *p* = 0.3067), *SLC44A4* (χ^2^ = 3.8182, *p* = 0.1482), *SLC35F3* (χ^2^ = 3.8537, *p* = 0.1456), and *SLC25A19* (χ^2^ = 5.0909, *p* = 0.0784).

Sex-related differences in the expression of the six genes were also analyzed. The Wilcoxon test, with an adjusted significance threshold of *p* < 0.0250, revealed no statistically significant differences between women and men in the expression of any of the genes in any of the tissue regions examined.

To investigate the relationships among genes involved in vitamin B_1_ metabolism, correlations between their expression levels were analyzed in glioblastoma tissue (Table 2). No significant correlation was observed between the expression of the two principal thiamine transporter genes, *SLC19A2* and *SLC19A3*. The correlation analysis indicated that the genes could be divided according to their relationships with these two principal thiamine transporters. *TPK1* expression correlated with *SLC19A2* expression but not with *SLC19A3* expression. A similar pattern was observed for the two less extensively characterized transporter genes, *SLC35F3* and *SLC44A4*, whose expression levels did not correlate with one another. *SLC35F3* expression correlated with *SLC19A2* but not with *SLC19A3*. Conversely, *SLC44A4* expression correlated with *SLC19A3* but not with *SLC19A2*. *SLC35F3* expression also correlated with *TPK1* expression but not with *SLC25A19* expression. By contrast, *SLC44A4* expression correlated with *SLC25A19* but not with *TPK1*. In the peritumoral area, expression of the two principal thiamine transporter genes, *SLC19A2* and *SLC19A3*, was significantly correlated. *TPK1* expression was also correlated with the expression of both transporter genes.

### 3.5. Vitamin B_1_ Metabolism in Glioblastoma Tissue Is Associated with Selected Patient Characteristics, Although Such Associations Are Infrequent

Correlations were calculated between the expression levels of genes involved in vitamin B_1_ metabolism in glioblastoma tissue and patient characteristics, including age at surgery, height, body weight, and BMI. The purpose of this analysis was to identify potential associations between vitamin B_1_ metabolism and the clinical or anthropometric characteristics of patients with glioblastoma.

The largest number of significant correlations was observed in the peritumoral area. Higher BMI was associated with lower expression of *SLC19A3* (*r_S_* = −0.46, *p* = 0.037) and *SLC25A19* (*r_S_* = −0.51, *p* = 0.017). Similarly, patient age was negatively correlated with the expression of *SLC19A2*, which encodes another major thiamine transporter (*r_S_* = −0.55, *p* = 0.011). Among men, *SLC19A2* expression in the peritumoral area was also positively correlated with height (*r_S_* = 0.68, *p* = 0.011). These findings suggest that age and anthropometric characteristics may be associated with thiamine transport and metabolism in peritumoral brain tissue.

Analysis of glioblastoma samples from all patients revealed only one significant association: *SLC44A4* expression in the tumor core was negatively correlated with body weight (*r_S_* = −0.48, *p* = 0.037) (Table 3). Additional correlations were identified when patients were analyzed separately according to sex. Among men, *SLC19A3* expression in the enhancing tumor region was positively correlated with height (*r_S_* = 0.70, *p* = 0.035). *SLC35F3* expression was positively correlated with BMI in the enhancing tumor region (*r_S_* = 0.73, *p* = 0.026) and with height in the tumor core (*r_S_* = 0.62, *p* = 0.031). These associations were not observed among women. Among women, *SLC25A19* expression in the tumor core was negatively correlated with age (*r_S_* = −0.74, *p* = 0.037). No corresponding association was observed among men.

Overall, the analysis indicates that the expression of genes involved in vitamin B_1_ metabolism is associated with patient characteristics in only a limited number of cases. Given the number of correlations tested and the relatively small sex-stratified subgroups, these findings should be interpreted cautiously.

### 3.6. Immunohistochemical Staining

**Figure 7:**
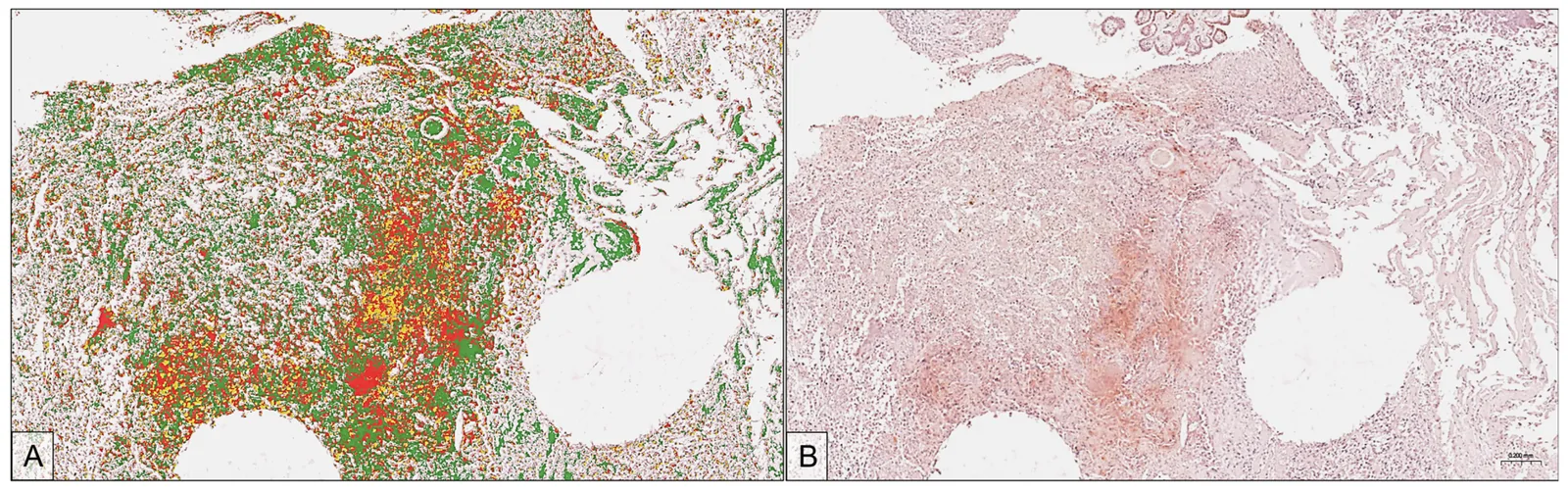
Immunohistochemical staining showing TPK1 expression in a glioblastoma sample obtained from the glioblastoma tumor. (**A**) Quantitative digital image analysis performed using Pattern Quant. Red areas indicate cytoplasmic regions positive for TPK1 immunostaining, green areas indicate cytoplasmic regions negative for TPK1 immunostaining, and yellow areas indicate immunonegative cell nuclei. (**B**) Representative image without Pattern Quant analysis, Scale = 0.200mm.

Immunohistochemical staining was performed to assess the distribution of TPK1 expression within the glioblastoma tumor (Figure 7). Overall, TPK1 expression was low; however, distinct areas with stronger immunoreactivity were observed within the tumor. High TPK1 expression was particularly evident in regions displaying a pseudopalisading pattern [71]. Pseudopalisades are characteristic histopathological structures in glioblastoma in which tumor cells accumulate around areas of hypoxia and necrosis.

## 4. Discussion

### 4.1. Increased Thiamine Availability Promotes Glioblastoma Cell Viability

Our results showed that thiamine concentrations of up to 2 µM increased the MTT metabolic activity of the glioblastoma cell lines analyzed by approximately 23% after six days of incubation. At higher concentrations, the effect of thiamine on cell viability gradually declined toward the control level. However, in LN-229 cells, millimolar thiamine concentrations continued to promote cell viability. To our knowledge, this is the first study to demonstrate that thiamine increases the viability of glioblastoma cells.

Our findings are consistent with previous studies of other tumor types. In an in vivo Ehrlich ascites tumor model, increased thiamine supplementation promoted tumor growth, with the maximum effect observed at a dose corresponding to 25 times the RDA. At 125 times the RDA, the tumor growth rate returned to the level observed in the control group [30]. This dose–response pattern is similar to that observed in the present study. The thiamine concentration in CSF is approximately 50 nM [19], and a 25-fold increase in this concentration corresponds to 1.25 µM. This value is close to the thiamine concentration at which the strongest increase in the viability of both glioblastoma cell lines was observed in our experiments. These findings suggest that physiological thiamine concentrations in blood and CSF do not support maximum viability of glioblastoma cells.

Thiamine is required for the activity of TKT [20], TKTL1 and TKTL2 [21], and OGDH [24]. TKT, TKTL1, and TKTL2 function in the non-oxidative branch of the pentose phosphate pathway, which contributes to the production of ribose 5-phosphate [22]. Ribose 5-phosphate is required for nucleotide and nucleic acid synthesis and is therefore particularly important for rapidly proliferating cells. OGDH is an enzyme of the tricarboxylic acid cycle that enables glutamine-derived 2-oxoglutarate to enter oxidative metabolism. This allows 2-oxoglutarate to be converted into metabolites used in the synthesis of cellular components required by proliferating cancer cells [34,35]. However, some cancer cells can metabolize 2-oxoglutarate through reductive carboxylation, also referred to as the reverse TCA cycle, thereby bypassing OGDH [35].

Thiamine is therefore required for several metabolic processes that support cancer cell proliferation. Nevertheless, its physiological concentration appears to be insufficient to support the maximum proliferation of glioblastoma cells. This observation may ultimately be relevant to the development of dietary and supplementation recommendations for patients with glioblastoma and potentially other cancers. However, it should be emphasized that a thiamine concentration approximately 40 times higher than that found in CSF increased glioblastoma cell viability by only approximately 23% after six days. Thus, even a substantial increase in thiamine availability may have only a modest effect on glioblastoma cell growth, and the clinical relevance of this finding remains to be established.

Our experiments also showed that millimolar thiamine concentrations began to reduce the MTT metabolic activity of T98G cells. This finding is consistent with previous reports. In vitro studies of cell lines derived from several cancer types have shown that thiamine inhibits cell viability after five days of incubation, with a half-maximal inhibitory concentration (IC_50_) of approximately 5 mM [31,32]. This effect has been attributed to the inhibition of PDK by high thiamine concentrations [31,32], leading to increased activity of the pyruvate dehydrogenase complex. The resulting metabolic shift may adversely affect cancer cell viability. The thiamine concentration in plasma is approximately 10 nM, whereas that in CSF is approximately 50 nM [19]. These concentrations are approximately 500,000-fold and 100,000-fold lower, respectively, than the concentrations at which thiamine toxicity has been observed. Such high concentrations are therefore unlikely to have practical therapeutic value, particularly because thiamine may exert cytotoxic effects on normal fibroblasts at concentrations as low as 0.5 mM [33].

In contrast to the response observed in T98G cells, millimolar thiamine concentrations promoted the viability of LN-229 cells. No previous studies appear to have reported a comparable effect. At such high concentrations, thiamine may exert nonspecific effects, and this observation is unlikely to have direct clinical relevance because of the potential toxicity of such concentrations to the human body [33].

We also investigated whether increased thiamine availability affected the response of glioblastoma cells to TMZ. Thiamine at a concentration of 2 µM had no clear effect on the activity of TMZ. TMZ reduced the viability of cells cultured both with and without additional thiamine in a similar concentration-dependent manner. However, cells treated with both thiamine and TMZ exhibited higher viability than cells treated with TMZ alone, suggesting that the viability-promoting effect of thiamine may occur independently of the anticancer activity of TMZ.

The principal mechanism of TMZ action is DNA alkylation [72,73]. One of the major mechanisms of resistance to TMZ involves O^6^-methylguanine-DNA methyltransferase (MGMT), which removes methyl groups from O^6^-methylguanine lesions [73,74,75]. This repair mechanism does not directly depend on ribose production by TPP-dependent enzymes [76], which may explain why thiamine availability did not substantially affect the response to TMZ.

Nevertheless, 200 µM TMZ reduced the viability of T98G cells cultured with additional thiamine but did not affect the viability of cells cultured without thiamine supplementation. Previous studies have shown that stimulation of oncogenic pathways may, under some conditions, sensitize cancer cells to anticancer agents [77]. However, the effect observed in the present study produced only a modest reduction in T98G cell viability. The clinical relevance of the interaction between thiamine and TMZ therefore appears limited, particularly because thiamine itself promoted glioblastoma cell growth.

### 4.2. Vitamin B_1_ Antimetabolites May Have Therapeutic Potential Against Glioblastoma but Do Not Enhance the Effects of TMZ

To our knowledge, this is the first study to investigate the effects of the vitamin B_1_ antimetabolites oxythiamine and amprolium on the viability of glioblastoma cells. Oxythiamine reduced the MTT metabolic activity of LN-229 cells at concentrations of 5 µM and above and that of T98G cells at concentrations of 10 µM and above. In T98G cells, the maximum reduction in viability was reached at approximately 50 µM oxythiamine, at which growth decreased to approximately 66% of the control value. In LN-229 cells, oxythiamine reduced viability in a concentration-dependent manner, reaching 56 ± 11% of the control value at the highest concentration tested.

Studies examining the effects of oxythiamine on cell lines derived from other tumor types support our findings. In Mia pancreatic adenocarcinoma cells, the IC_50_ of oxythiamine was reported to be 14.95 µM [46]. In our experiments, half of the maximum reduction in viability occurred at concentrations between 10 and 20 µM, which is consistent with the value reported in that study.

Our results indicate that oxythiamine reduces glioblastoma cell viability only to a certain level, corresponding to approximately 60% of the control value. Similar plateaus have been observed in some studies of other cancer types. In A549 cells, oxythiamine reduced viability to a minimum of approximately 70% of the control value [47], whereas in HeLa cell growth decreased to 40 ± 16% of the control value [43,78]. However, sensitivity to oxythiamine varies among cell lines. In the SKOV3 and OV90 ovarian cancer cell lines, its effects became apparent only at 500 µM [79]. Conversely, in Mia pancreatic adenocarcinoma cells, oxythiamine reduced viability to almost zero at a concentration of only 40 µM [46].

In T98G cells, an additional reduction in growth was observed at the highest oxythiamine concentration tested, 1 mM, compared with the effects observed at lower concentrations. This may be associated with the inhibition of additional enzymes at very high oxythiamine concentrations. One possible target is TPK1, the activity of which is inhibited by oxythiamine at a concentration of 4.2 mM [45].

The anticancer properties of oxythiamine are generally attributed to the inhibition of transketolase activity [80,81]. Transketolases function in the non-oxidative branch of the pentose phosphate pathway, which contributes to the production of ribose 5-phosphate required for DNA synthesis and cancer cell proliferation [82]. This mechanism is consistent with our finding that oxythiamine reduces the viability of glioblastoma cells. However, ribose 5-phosphate can also be synthesized through the oxidative branch of the pentose phosphate pathway, independently of transketolase activity [81]. This alternative pathway may explain why oxythiamine reduced the viability of the analyzed cells only to a limited extent.

To our knowledge, the present study is also the first to investigate amprolium as a potential therapeutic agent against glioblastoma. Amprolium showed modest anticancer activity against the glioblastoma cell lines analyzed. It reduced LN-229 cell viability at concentrations of 50 µM and above. At 100 µM, viability reached a minimum of approximately 93% of the control value and did not decrease further with increasing amprolium concentrations. In T98G cells, viability decreased progressively at concentrations of 50 µM and above, reaching 75 ± 6% of the control value at the highest concentration tested.

No previous studies appear to have directly investigated the effects of amprolium on cancer cell viability. Our findings can, however, be considered in relation to the biochemical properties of this antimetabolite. Amprolium inhibits TPK1 activity at a concentration of approximately 180 µM [45], which is within the same concentration range as that at which anticancer effects were observed in our experiments.

At high concentrations, amprolium produced a smaller reduction in cell growth than oxythiamine. Within the concentration range tested, amprolium acts at an early stage of thiamine metabolism by inhibiting TPK1 [45]. Oxythiamine, by contrast, primarily inhibits downstream TPP-dependent enzymes such as transketolases and does not substantially inhibit TPK1 at the concentrations used in our experiments [45,80,81]. It is therefore possible that intracellular thiamine metabolites and the products of TPP-dependent enzymes that had accumulated before or during exposure to amprolium were sufficient to support glioblastoma cells throughout the incubation period. Extending the incubation time by 24 h produced a further reduction in cell viability. In T98G cells, 100 µM oxythiamine reduced viability from 66 ± 5% to 48 ± 2% (*p* = 0.00117), whereas 200 µM amprolium reduced viability from 83 ± 6% to 76 ± 5%. This time-dependent effect may reflect the gradual depletion of intracellular metabolites or the cumulative action of the vitamin B_1_ antimetabolites on proliferating cells.

To determine whether vitamin B_1_ antimetabolites could be incorporated into standard glioblastoma treatment, we examined their interaction with TMZ, the chemotherapeutic drug routinely used in this disease. The effect of TMZ alone on LN-229 and T98G cell viability was first assessed. At 500 µM, TMZ reduced LN-229 cell viability to 80 ± 11% and T98G cell viability to 73 ± 6% relative to the corresponding DMSO controls. These results are broadly consistent with previous reports. T98G is a glioblastoma cell line characterized by high MGMT expression [83], which contributes to its marked resistance to TMZ. Depending on the study, the reported IC_50_ of TMZ in T98G cells ranges from 418 µM [84] and approximately 800 µM [85] to as high as 2 mM [86]. For LN-229 cells, reported TMZ IC_50_ values range from approximately 1 to 2 mM [87]. Such high IC_50_ values may be difficult to achieve experimentally and may be affected by methodological limitations. TMZ is poorly soluble in aqueous solutions but dissolves more readily in DMSO [88]. Nevertheless, its solubility in DMSO is not unlimited. At very high TMZ concentrations, the amount of DMSO added to the culture medium may become substantial and may itself affect the analyzed cells. All TMZ results in the present study were therefore expressed relative to the corresponding DMSO controls. At the concentrations used, DMSO did not affect LN-229 cells but exerted a slight cytotoxic effect on T98G cells.

After assessing the effects of TMZ alone, we investigated its interaction with the vitamin B_1_ antimetabolites. The tested compounds did not enhance the reduction in cell viability produced by TMZ. On the contrary, under the experimental conditions used, the reduction in viability was smaller after combined treatment than after treatment with TMZ alone. To our knowledge, this is the first study to examine the combined effects of vitamin B_1_ antimetabolites and TMZ. Direct comparison with previous studies is therefore not possible. However, interactions between oxythiamine and other chemotherapeutic agents have been investigated. Oxythiamine has been reported to enhance the effects of cisplatin in a cervical cancer model [89]. This effect has been associated with the inhibition of transketolase activity, as reduced transketolase activity increases the sensitivity of cervical cancer cells to cisplatin [90]. TMZ resistance, however, may not require transketolase activity and may therefore be largely independent of thiamine metabolism. TMZ is a DNA-alkylating agent that produces predominantly N^7^-methylguanine, together with other DNA base modifications, particularly N^3^-methyladenine and O^6^-methylguanine [72,73]. O^6^-methylguanine lesions are removed by MGMT through direct transfer of the methyl group [73,75]. This is one of the principal mechanisms of TMZ resistance [73,74,75] and does not directly depend on ribose production [76]. This may explain why the vitamin B_1_ antimetabolites did not enhance the effects of TMZ in our experiments.

### 4.3. Chronic Hypoxia Suppresses Vitamin B_1_ Homeostasis

This is the first study to investigate the expression of *SLC35F3* and *SLC44A4* in glioblastoma cells. qRT-PCR analysis showed no detectable expression of *SLC35F3* or *SLC44A4* in LN-229 cells, while *SLC35F3* expression was not detectable in T98G cells. Comparison with the available literature is difficult because data on the role of these two transporters in cancer are scarce. *SLC35F3* has been reported to have a tumor-promoting role in lung adenocarcinoma [91], but no data are currently available on the significance of either *SLC35F3* or *SLC44A4* in glioblastoma. Nevertheless, our findings in LN-229 and T98G cells are consistent with our results obtained in glioblastoma tumor tissue, in which the expression of both transporters was reduced. Taken together, these findings suggest that glioblastoma cells, particularly malignant cells within the tumor, may exhibit low or undetectable expression of *SLC35F3* and *SLC44A4*.

The effect of TMZ on vitamin B_1_ homeostasis in cancer cells has not been investigated previously. In our study, compared with the DMSO-treated control, TMZ did not significantly affect the expression of genes involved in vitamin B_1_ homeostasis. However, these findings are substantially confounded by the effects of DMSO on the parameters examined. Although no previous studies have directly examined the effect of TMZ on this process, studies of other anticancer drugs have shown that doxorubicin and cisplatin increase *TPK1* expression [92]. Increased *TPK1* expression may enhance the production of TPP, a cofactor required by enzymes involved in ribose synthesis. Ribose, in turn, provides a precursor for deoxyribose synthesis and thus supports DNA repair [93]. TMZ acts through a different mechanism. As a DNA-alkylating agent, it induces the formation of O^6^-methylguanine lesions in DNA [72,73]. These lesions can be repaired directly by MGMT [73,75,76]. Therefore, resistance to TMZ may not require increased activity of thiamine-dependent pathways involved in ribose production to the same extent as resistance to agents that induce other types of DNA damage. This may partly explain the lack of a clear effect of TMZ on genes involved in vitamin B_1_ homeostasis in the glioblastoma cells examined.

It should also be noted that both LN-229 [87] and T98G [83,84,86] are relatively resistant to TMZ. Consequently, relatively high TMZ concentrations are required to produce a pronounced response in these cells. TMZ is commonly dissolved in DMSO, but its solubility in this solvent is limited. Thus, the use of higher TMZ concentrations also requires higher DMSO concentrations. Because DMSO itself may affect cellular processes and gene expression, this introduces a potential confounding factor and makes it more difficult to distinguish the specific effects of TMZ from those of the solvent.

No previous study has examined the effect of chronic hypoxia on the expression of genes involved in vitamin B_1_ homeostasis in glioblastoma cells. Overall, chronic hypoxia tended to reduce the expression of the genes examined, although the response differed between the two cell lines. In T98G cells, chronic hypoxia significantly decreased *SLC44A4* expression, whereas no significant change in this gene was observed in LN-229 cells. Conversely, chronic hypoxia significantly decreased *SLC19A3* and *TPK1* expression in LN-229 cells. These findings are partly consistent with previous studies and suggest that the effects of chronic hypoxia on individual components of vitamin B_1_ homeostasis are strongly dependent on the cellular model.

The effect of chronic hypoxia on *TPK1* expression also appears to be model dependent. Chronic hypoxia has been reported to increase *TPK1* expression in colorectal carcinoma cells [92], whereas decreased expression was observed in PC-3 prostate cancer cells [94]. In our study, chronic hypoxia also decreased *TPK1* expression in LN-229 cells.

In contrast, the available data concerning *SLC19A2* are more consistent across different cancer models. Chronic hypoxia did not affect *SLC19A2* expression in BT474 breast cancer cells [95], SK-N-BE neuroblastoma cells [96], PC-3 prostate cancer cells, WM793B melanoma cells, or SK-OV-3 ovarian cancer cells [94]. These findings are consistent with our results in both LN-229 and T98G cells, in which no significant effect of chronic hypoxia on *SLC19A2* expression was observed.

The response of *SLC19A3* to chronic hypoxia appears to be more variable. Chronic hypoxia increased *SLC19A3* expression in BT474 breast cancer cells [95] and SK-N-BE neuroblastoma cells [96], whereas no significant effect was observed in PC-3 prostate cancer cells, WM793B melanoma cells, or SK-OV-3 ovarian cancer cells [94]. In the present study, chronic hypoxia decreased *SLC19A3* expression in LN-229 cells. A decrease was also observed in T98G cells, although it did not reach statistical significance.

Chronic hypoxia increases *SLC44A4* expression in prostate cancer cells [94]. However, this effect depends on the cancer type, as chronic hypoxia does not alter the expression of this gene in neuroblastoma or ovarian cancer cells [94]. In our study, chronic hypoxia decreased *SLC44A4* expression in T98G cells but not in LN-229 cells, indicating that the effect is cell line-dependent.

### 4.4. Expression of Genes Involved in Thiamine Metabolism Is Lower in Glioblastoma Tissue than in the Peritumoral Area

Our results showed that the expression of *SLC19A3*, *TPK1*, *SLC25A19*, *SLC35F3*, and *SLC44A4* was lower in glioblastoma tissue than in the peritumoral area. No corresponding difference was observed for *SLC19A2*, which encodes another thiamine transporter. To our knowledge, this is the first study to analyze the expression of genes involved in vitamin B_1_ metabolism in glioblastoma tissue. Direct comparisons with previous studies of glioblastoma are therefore not possible. However, our findings can be considered in the context of results obtained for other cancer types.

*SLC19A3* expression is lower in breast cancer [13,97] and gastric cancer [98] tissues than in the corresponding non-malignant tissues. This reduction has been associated with hypermethylation of the *SLC19A3* promoter and may be consistent with the lower *SLC19A3* expression observed in the present study. However, reduced expression of this transporter is not a universal feature of cancer. In cervical cancer, *SLC19A3* expression is higher than in non-malignant tissue [99]. By contrast, *SLC19A2* expression does not differ between breast cancer and non-malignant breast tissue [13], which is consistent with our findings in glioblastoma.

The other genes analyzed included *TPK1* and *SLC25A19*. In breast cancer, *TPK1* expression is higher than in non-malignant tissue [100]. Similarly, increased expression of *SLC25A19*, which encodes a mitochondrial TPP transporter, has been reported in breast cancer [100], colorectal cancer [101], and hepatocellular carcinoma [102]. These findings differ from those of the present study, in which *TPK1* and *SLC25A19* expression was lower in glioblastoma tissue than in the peritumoral area. The regulation of these genes may therefore depend on tumor type and its specific metabolic requirements.

We also examined the less extensively characterized transporters SLC35F3 and SLC44A4, which are involved in vitamin B_1_ metabolism. To our knowledge, this is the first study to show that *SLC35F3* and *SLC44A4* expression is lower in glioblastoma tissue than in the peritumoral area. Both transporters remain poorly characterized in cancer. *SLC35F3* expression is increased in lung adenocarcinoma compared with non-malignant tissue [91], which contrasts with our results. This further suggests that the regulation of this transporter may be tumor-type specific. Conversely, *SLC44A4* expression is lower in colorectal cancer than in non-malignant tissue [103], which is consistent with our observations in glioblastoma.

Overall, some genes involved in vitamin B_1_ metabolism appear to be downregulated in glioblastoma in a manner similar to that observed in certain other cancers. However, the expression patterns of the remaining genes may depend closely on tumor type and may therefore differ substantially between glioblastoma and other malignancies.

Correlation analysis revealed close associations between the expression levels of selected genes involved in vitamin B_1_ metabolism. In glioblastoma tissue, no significant correlation was observed between the expression of the two major thiamine transporter genes, *SLC19A2* and *SLC19A3* [13]. By contrast, *SLC19A2* expression correlated with the expression of *SLC35F3* and *TPK1*, whereas *SLC19A3* expression correlated with *SLC44A4* expression. These findings suggest the presence of two expression-related groups: one comprising *SLC19A2*, *SLC35F3*, and *TPK1*, and another comprising *SLC19A3* and *SLC44A4*.

To our knowledge, this is also the first study to examine associations between the expression of genes involved in thiamine metabolism and patient characteristics in glioblastoma samples. In the peritumoral area, BMI was negatively correlated with the expression of *SLC19A3* and *SLC25A19*. SLC19A2 and SLC19A3 are among the principal thiamine transporters, although SLC19A3 exhibits more favorable thiamine transport kinetics than SLC19A2 [13]. The peritumoral area is commonly used as a reference tissue in glioblastoma studies [62]. Nevertheless, it should not be regarded as fully equivalent to healthy brain tissue. The observed correlations may suggest that greater BMI is associated with reduced expression of genes involved in cellular thiamine uptake and mitochondrial TPP transport in peritumoral tissue. TPP is a cofactor for the pyruvate dehydrogenase complex [23], OGDH, an enzyme of the TCA cycle [24], and BCKDH, which participates in the catabolism of branched-chain amino acids [25]. However, gene expression alone does not establish reduced transporter activity, impaired cerebral energy metabolism, or altered amino acid catabolism.

No previous studies appear to have investigated associations between BMI and the expression of genes involved in vitamin B_1_ metabolism in human brain tissue. However, an inverse association between BMI and *SLC19A3* expression has been reported in human subcutaneous adipose tissue [104]. This finding provides limited support for a broader relationship between BMI and the regulation of *SLC19A3* expression.

We also identified a negative correlation between *SLC19A2* expression and patient age in the peritumoral area. This association may indicate that the expression of this thiamine transporter declines with age in peritumoral brain tissue. Because thiamine is required for the activity of TPP-dependent enzymes, including the PDC [23] and OGDH [24], reduced thiamine transport could potentially affect cellular energy metabolism. Nevertheless, the present cross-sectional correlation does not demonstrate an age-related decline in cerebral thiamine metabolism or establish its functional consequences. Similar caution applies to the correlations observed for BMI.

Analysis of glioblastoma samples revealed the following associations:

- A positive correlation between *SLC19A3* expression and height among men;
- A negative correlation between *SLC25A19* expression and age at surgery among women;
- A negative correlation between *SLC44A4* expression and body weight in the entire study group;
- Positive correlations between *SLC35F3* expression and height or BMI among men, depending on the tumor region analyzed.

SLC25A19 is a mitochondrial TPP transporter [18]. Because TPP is required for OGDH activity in the TCA cycle [24], the negative association between *SLC25A19* expression and age among women might reflect age-related differences in mitochondrial thiamine metabolism in glioblastoma cells. However, OGDH activity was not measured, and this mechanistic interpretation remains speculative.

The roles of SLC35F3 and SLC44A4 in cancer remain poorly understood. SLC35F3 is a thiamine transporter [15], whereas SLC44A4 transports TPP and acetylcholine but not unphosphorylated thiamine [16]. The observed positive correlations with height among men may indicate differences in transporter-gene expression associated with anthropometric characteristics. However, they do not demonstrate increased thiamine uptake by glioblastoma cells or an effect on tumor growth. Given the small sex-stratified subgroups and the number of correlations tested, these findings require confirmation in larger patient cohorts.

### 4.5. Glioblastoma Contains Regions with High TPK1 Protein Expression

Immunohistochemical staining of glioblastoma tissue samples revealed regions with high TPK1 expression. Glioblastoma is a heterogeneous tumor containing structures known as pseudopalisades [71]. These structures have hypoxic centers surrounded by proliferating cancer cells. Vitamin B_1_ metabolism is likely to be particularly active in these proliferative regions. However, more detailed studies are needed to understand glioblastoma as a whole, particularly the role of vitamin B_1_ in tumor function. In this study, we sought to investigate the importance of vitamin B_1_ for cancer cells. Our findings provide a starting point for further research on this topic.

## 5. Conclusions: Clinical Significance of the Findings

In the present study, vitamin B_1_ antimetabolites were investigated as potential therapeutic agents against glioblastoma. Such compounds have previously been considered potential anticancer agents, with oxythiamine being the most extensively studied. In vitro anticancer activity of oxythiamine has been reported in cervical carcinoma [78], hepatocellular carcinoma [105], and thyroid cancer [106]. Antitumor effects have also been observed in in vivo models of Ehrlich ascites tumor [22,30], Lewis lung carcinoma [107], and human papillomavirus (HPV)-associated cervical cancer [89]. In a clinical study, benfo-oxythiamine was reported to reduce radioresistance in patients with metastatic castration-resistant prostate cancer, thereby improving the effects of treatment with the radiopharmaceutical ^177^Lu-PSMA-I&T [108]. These findings indicate that interference with thiamine-dependent metabolism may have therapeutic value in selected cancer settings.

In our experiments, vitamin B_1_ antimetabolites reduced glioblastoma cell viability only to a limited extent after 72 h of incubation. Longer exposure, including the preincubation period, produced a greater reduction in viability, indicating that glioblastoma cells are partly dependent on thiamine metabolism. Nevertheless, the antimetabolites reduced cell viability by no more than approximately 40% after 72 h. Their relatively modest effect despite prolonged exposure suggests that these compounds may have limited value as stand-alone treatments for glioblastoma. Vitamin B_1_ metabolism is also essential for normal human cells. Prolonged systemic inhibition of this pathway could therefore produce adverse effects that outweigh its antitumor activity. The therapeutic window, selectivity, pharmacokinetics, and toxicity of the compounds would need to be established before their potential clinical application could be assessed.

The blood–brain barrier (BBB), which limits the exposure of glioblastoma cells to hydrophilic drugs, must also be considered [109]. Vitamin B_1_ antimetabolites are hydrophilic compounds. Therefore, further studies are needed to determine whether, and to what extent, they cross the BBB and reach glioblastoma tissue.

We also investigated whether oxythiamine and amprolium enhanced the anticancer activity of TMZ, which is routinely used in the treatment of glioblastoma. No evidence of a beneficial interaction was observed. Instead, the tested antimetabolites appeared to reduce the effect of TMZ under the experimental conditions used. Consequently, combining these vitamin B_1_ antimetabolites with TMZ is unlikely to improve the therapeutic efficacy of TMZ.

The present study did not evaluate combinations of the antimetabolites with radiotherapy or with anticancer drugs other than TMZ. Oxythiamine has been reported to reduce resistance to cisplatin [89] and radiotherapy [108], thereby increasing the effectiveness of these treatments. It is therefore possible that compounds targeting thiamine metabolism could be useful in other combination strategies. However, their combination with TMZ does not appear promising based on the present findings.

We further showed that increasing the concentration of thiamine in the culture medium promoted glioblastoma cell viability. This observation may have clinical implications. High doses of TPP have been proposed as a means of reducing the adverse effects of chemotherapy [110,111,112,113]. However, some reported protective effects may partly result from interactions between thiamine and anticancer drugs that reduce the activity of the drugs [114]. Our findings raise the additional possibility that increased thiamine availability may support cancer cell proliferation. Nevertheless, this effect was demonstrated only in cultured glioblastoma cells and should not be directly extrapolated to patients. Further studies are needed to determine whether high thiamine or TPP concentrations interact with commonly used chemotherapeutic agents or radiotherapy and whether they affect tumor growth in vivo. Conversely, thiamine deficiency and Wernicke encephalopathy may occur in patients with cancer [115,116]. In such cases, thiamine administration is medically necessary and should not be withheld on the basis of in vitro findings. The treatment of a clinically relevant deficiency must take precedence over theoretical concerns regarding tumor growth.

The viability-promoting effect of increased thiamine concentrations may also be relevant to dietary counseling for patients with glioblastoma, including after treatment. However, the present results do not justify dietary thiamine restriction, because the experiments were performed in vitro and thiamine is an essential nutrient. They may instead support avoiding unnecessary high-dose supplementation in patients without a diagnosed deficiency until further clinical evidence becomes available.

Data from the Gene Expression Profiling Interactive Analysis (GEPIA) platform [117] indicate that higher *TPK1* and *SLC44A4* expression in glioblastoma is associated with poorer survival, whereas higher *SLC19A2* expression is associated with better survival. GEPIA analyzes data from multiple cancer types available through The Cancer Genome Atlas [118] together with non-malignant tissue data from the Genotype-Tissue Expression database [119,120].

TPK1 plays a central role in vitamin B_1_ metabolism by converting thiamine into TPP, the active cofactor required by TPP-dependent enzymes [17]. The associations observed in GEPIA suggest that genes involved in thiamine metabolism may have prognostic relevance in glioblastoma and could represent potential therapeutic targets. However, these database-derived associations do not establish causality. Moreover, the results of the present study indicate that the vitamin B_1_ antimetabolites tested are unlikely to be effective when combined with TMZ.

## Figures and Tables

**Table 1:** Patient characteristics.

Characteristic	Mean	SD
Age at surgery [y]	60.7	12.5
Weight [kg]	84	19
Height [cm]	172	12
Body mass index (BMI) kg/m^2^)	28.7	4.8
Sex	12 women and 18 men	

**Table 2:** Spearman correlations among the expression levels of genes involved in thiamine metabolism across the analyzed tissue regions.

Studied Gene/Location		*SLC19A3*			*TPK1*			*SLC19A2*			*SLC44A4*			*SLC35F3*			*SLC25A19*		
		PA	ETR	TC	PA	ETR	TC	PA	ETR	TC	PA	ETR	TC	PA	ETR	TC	PA	ETR	TC
*SLC19A3*	PA	1.00																	
	ETR	−0.12	1.00																
	TC	0.44	−0.19	1.00															
*TPK1*	PA	0.54 *	−0.28	0.20	1.00														
	ETR	0.18	0.25	0.31	−0.04	1.00													
	TC	0.11	0.04	0.25	0.17	0.22	1.00												
*SLC19A2*	PA	0.42	−0.37	0.22	0.68 *	0.15	0.21	1.00											
	ETR	−0.09	0.36	0.08	−0.16	0.82 *	0.24	0.06	1.00										
	TC	0.18	−0.09	0.28	0.35	0.08	0.67 *	0.33	0.08	1.00									
*SLC44A4*	PA	0.98 *	−0.12	0.49	0.51 *	0.24	0.15	0.40	−0.09	0.22	1.00								
	ETR	−0.03	0.92 *	−0.23	−0.12	0.22	0.11	−0.21	0.27	−0.01	−0.03	1.00							
	TC	0.49 *	−0.15	0.94 *	0.21	0.24	0.20	0.14	0.04	0.19	0.51 *	−0.21	1.00						
*SLC35F3*	PA	0.02	−0.05	−0.20	0.51 *	−0.10	0.12	0.49	0.08	0.01	−0.06	0.02	−0.12	1.00					
	ETR	0.14	0.21	0.16	−0.02	0.58 *	0.17	0.15	0.58 *	0.19	0.17	0.10	0.19	0.16	1.00				
	TC	−0.06	0.40	0.26	−0.19	0.43 *	0.58 *	−0.08	0.48 *	0.47 *	−0.03	0.31	0.16	−0.06	0.37	1.00			
*SLC25A19*	PA	0.61 *	−0.25	0.13	0.60 *	−0.01	0.19	0.58 *	−0.13	0.32	0.60 *	−0.14	0.09	0.07	−0.08	−0.20	1.00		
	ETR	−0.19	0.41	−0.02	−0.25	0.70 *	0.29	−0.06	0.65 *	0.08	−0.16	0.50	−0.10	−0.04	0.28	0.45	−0.15	1.00	
	TC	0.57 *	−0.14	0.47	0.25	0.14	0.43	0.33	0.08	0.46	0.58 *	−0.14	0.48	−0.05	0.15	0.15	0.62 *	−0.06	1.00

Spearman’s rank correlation coefficients with the correction of the FDR *p*-value (False Discovery Rate) according to the Benjamini–Hochberg procedure (BH procedure) are presented. *—*p* < 0.05; PA—peritumoral area; ETR—enhancing tumor region; TC—tumor core.

**Table 3:** Correlations between the expression levels of the analyzed genes in different tumor regions and patient age and anthropometric characteristics in the entire study group and in women and men analyzed separately.

Studied Gene	Location	Age at Surgery	Weight	Height	BMI	Age at Surgery	Weight	Height	BMI	Age at Surgery	Weight	Height	BMI
		All Patients				Women				Men			
*SLC19A3*	PA	−0.19	−0.31	0.12	−0.46 *	0.06	−0.72 *	−0.35	−0.48	−0.15	−0.35	0.18	−0.55
	ETR	−0.38	−0.08	0.16	−0.25	−0.35	−0.13	−0.08	0.00	−0.30	0.34	0.70 *	−0.20
	TC	−0.42	−0.40	−0.03	−0.42	−0.50	−0.36	−0.05	−0.33	−0.36	−0.26	0.25	−0.38
*TPK1*	PA	−0.39	0.00	0.24	−0.14	−0.31	−0.12	0.30	−0.31	−0.46	0.11	0.46	−0.20
	ETR	−0.11	0.15	−0.06	0.32	−0.18	0.45	0.10	0.32	−0.07	0.18	−0.04	0.32
	TC	−0.31	−0.15	0.08	−0.33	−0.57	−0.49	−0.17	−0.31	−0.13	−0.08	0.20	−0.36
*SLC19A2*	PA	−0.55 *	0.02	0.33	−0.17	−0.48	−0.26	0.37	−0.52	−0.54	0.21	0.68 *	−0.10
	ETR	−0.35	0.09	0.05	0.07	−0.57	−0.13	0.44	−0.38	−0.22	0.40	0.20	0.42
	TC	−0.30	0.21	0.21	0.13	−0.40	0.42	0.33	0.12	−0.23	0.14	0.28	0.02
*SLC44A4*	PA	−0.27	−0.19	0.23	−0.38	−0.17	−0.50	−0.02	−0.43	−0.20	−0.28	0.21	−0.50
	ETR	−0.28	−0.15	0.08	−0.24	−0.20	−0.24	−0.20	−0.05	−0.32	−0.03	0.45	−0.43
	TC	−0.28	−0.48 *	−0.15	0.39	−0.50	−0.36	−0.05	−0.33	−0.09	−0.34	−0.01	−0.34
*SLC35F3*	PA	−0.09	−0.04	−0.02	−0.03	0.05	−0.27	−0.07	−0.26	−0.09	0.20	0.14	0.17
	ETR	−0.07	0.16	0.03	0.14	−0.15	0.19	0.48	−0.10	0.12	0.63	−0.17	0.73 *
	TC	−0.04	−0.04	−0.06	−0.06	−0.01	−0.13	−0.30	0.06	−0.03	0.52	0.62 *	0.16
*SLC25A19*	PA	−0.43	−0.28	0.25	−0.51 *	−0.40	−0.83 *	−0.18	−0.74 *	−0.37	−0.41	0.21	−0.64 *
	ETR	−0.18	0.14	0.02	0.25	−0.66	−0.06	0.17	−0.22	0.10	0.29	−0.13	0.37
	TC	−0.34	−0.36	0.02	−0.44	−0.74 *	−0.51	0.24	−0.62	−0.04	−0.47	0.07	−0.50

Spearman’s rank correlation coefficients are presented. An asterisk indicates statistical significance at *p* < 0.05. PA—peritumoral area; ETR—enhancing tumor region; TC—tumor core.

## Data Availability

The original data presented in the study are openly available in RepOD at https://repod.icm.edu.pl/dataset.xhtml?persistentId=doi:10.18150/UIIBBE (accessed on 1 September 2026).

## Supplementary Materials

The following supporting information can be downloaded at: https://www.mdpi.com/article/10.3390/cancers18213423/s1. File S1: Original WB plot.

## Abbreviations

ATCC	American Type Culture Collection
BBB	Blood–brain barrier
BCKDH	Branched-chain α-keto acid dehydrogenase
BER	Base excision repair
BMI	Body mass index
CSF	Cerebrospinal fluid
DHTKD1	Dehydrogenase E1 and transketolase domain-containing protein 1
DMEM	Dulbecco′s modified eagle′s medium
DMSO	Dimethyl sulfoxide
DPBS	Dulbecco’s phosphate-buffered saline
FDR	False discovery rate
EDTA	Ethylenedinitrilotetraacetic acid
ETR	Enhancing tumor region
FBS	Fetal bovine serum
GAPDH	Glyceraldehyde-3-phosphate dehydrogenase
GEPIA	Gene Expression Profiling Interactive Analysis
HACL1	2-hydroxyacyl-CoA lyase 1
HIF-1α	Hypoxia-inducible factor-1α
HPV	Human papillomavirus
IC_50_	Half-maximal inhibitory concentration
MEM	Minimum essential medium
MGMT	O^6^-methylguanine-DNA methyltransferase
MTT	3-(4,5-dimethylthiazol-2-yl)-2,5-diphenyltetrazolium bromide
OGDH	2-oxoglutarate dehydrogenase
PA	Peritumoral area
PDC	Pyruvate dehydrogenase complex
PDK	Pyruvate dehydrogenase kinase
PVDF	Polyvinylidene difluoride membranes
qRT-PCR	Quantitative reverse transcription real-time polymerase chain reaction
RDA	Recommended dietary allowance
RIPA	Radioimmunoprecipitation assay buffer
*r_S_*	Spearman’s rank correlation coefficient
SDS-PAGE	Sodium dodecyl sulfate-polyacrylamide gel electrophoresis
SLC19A2	Solute carrier family 19 member 2
SLC19A3	Solute carrier family 19 member 3
SLC22A1	Solute carrier family 22 member 1
SLC22A2	Solute carrier family 22 member 2
SLC25A19	Solute carrier family 25 member 19
SLC35A3	Solute carrier family 35 member 3
SLC44A4	Solute carrier family 44 member 4
TBP	TATA-box binding protein
TBS	Tris-buffered saline
TC	Tumor core
TCA	Tricarboxylic acid
TKT	Transketolase
TKTL1	Transketolase-like protein 1
TKTL2	Transketolase-like protein 2
TMZ	Temozolomide
TPK1	Thiamin pyrophosphokinase 1
TPP	Thiamine pyrophosphate
